# *Taraxacum sinicum* Kitag. (Binpu-3) root extract inhibits tumor invasion via Notch signaling in *Drosophila* and human breast cancer MDA-MB-231 cells

**DOI:** 10.3389/fphar.2025.1494545

**Published:** 2025-03-13

**Authors:** Jiawei Wu, Jianbo Zhang, Wanyu Shu, Wei Feng, Ran Meng, Lingyu Kong, Huijuan Cao, Chunhua Jiang, Sitong Wang, Fanwu Wu, Chenxi Wu, Xiuping Wang

**Affiliations:** ^1^ Hebei Key Laboratory of Integrated Traditional Chinese and Western Medicine for Diabetes and Its Complications, College of Traditional Chinese Medicine, North China University of Science and Technology, Tangshan, China; ^2^ Institute of Coastal Agriculture, Hebei Academy of Agriculture and Forestry Sciences, Tangshan, China; ^3^ Oncology of Chinese and Western Medicine, North China University of Science and Technology Affiliated Hospital, Tangshan, China; ^4^ Hebei Key Laboratory of Medical Engineering and Integrated Utilization of Saline Alkali Land, Hebei Administration of TCM Key Laboratory of Quality Control of Salt Alkali Resistant TCM, Tangshan, China

**Keywords:** *Taraxacum sinicum* Kitag., root extract, tumor invasion, Notch, *Drosophila*, breast cancer

## Abstract

Metastasis is the primary cause of death in patients with malignant tumors. Therefore, effectively controlling or reversing tumor cell growth and metastasis is crucial for treating malignant tumors. In this study, we investigated the effects and underlying mechanisms of Binpu-3 (a strain of *Taraxacum sinicum* Kitag., which was cultivated in slightly saline-alkali soil) on tumor invasion both in *Drosophila* and human breast cancer cells. High-performance liquid chromatography (HPLC) analysis revealed that caftaric, chlorogenic, caffeic, and cichoric acids in the Binpu-3 leaves and roots were significantly higher than those in the wild-type Handan strain. Binpu-3 root extract (Binpu-3RE) suppressed the invasion rate of tumor cells at 25.00 mg/mL in the *Drosophila eyeful* model, whereas Binpu-3 leaf extract had no obvious effect on tumor metastasis. Accordingly, we found that caffeic acid, quercetin, apigenin, and taraxasterol content in Binpu-3 roots was significantly higher than that in the leaves. In addition, ultra performance liquid chromatography-high resolution mass spectrometry (UPLC-HRMS) analysis revealed that Binpu-3RE contained various constituents, including pantothenate (0.1%), butein (0.53%), chlorogenate (0.78%), chicoric acid (1.96%), azelaic acid (0.23%), and [6]-gingerol (0.13%). *In vivo*, Binpu-3RE impeded *ptc*>*scrib-IR* triggered cell migration in *Drosophila* at an appropriate concentration, and 25.00 mg/mL was selected as the best dose to carry out follow-up mechanistic research. This dose of Binpu-3RE reduced the mRNA levels of Notch pathway key genes *Delta*, *Serrate*, *Notch*, *Su(H)*, and *En(spl)*, the expression levels of *NRE*-GFP (Notch activity reporter), β-integrin, and metalloproteinase-1 (MMP1) in *Drosophila*. Cell viability, wound healing, transwell, and Western blotting assays data implied that Binpu-3RE reduced cell growth, migration, invasion, and the expression of Notch1, Jagged1, and HES1 in human breast cancer MDA-MB-231 cells. In summary, the saline-alkali tolerant dandelion Binpu-3 used in this study was of excellent quality, and the root extract showed significant anti-tumor metastasis effects via reduction of Notch signal activity and the expression β-integrin and MMP1 proteins in *Drosophila* and breast cancer cells, providing a theoretical basis for the development and use of alkaline-soil dandelion herbs, and a therapeutic strategy for the clinical treatment of malignant breast cancer.

## Graphical Abstract

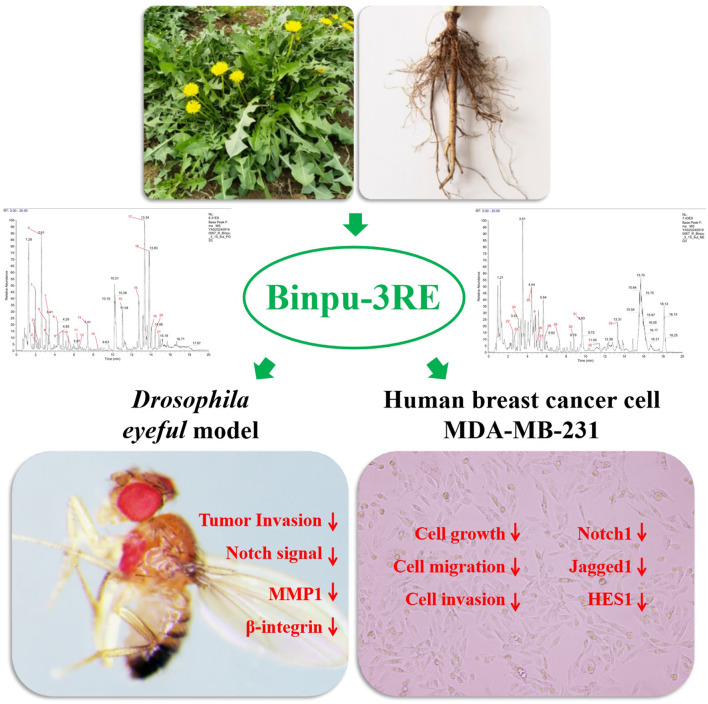

## Highlights

• Caftaric, chlorogenic, caffeic, and cichoric acid levels were high in Binpu-3.
• Binpu-3 root extract suppressed tumour invasion in the *Drosophila eyeful model*.
• Binpu-3RE inhibited *ptc*>*scrib-IR*-induced cell migration.
• 25.00 mg/ml of Binpu-3RE reduced Notch pathway-related gene expression in fly.
• Binpu-3RE impeded growth, migration, invasion, and Notch signal in human breast cancer MDA-MB-231 cells.

## 1 Introduction

Tumors are a serious threat to human health. As of 2023, more than 20 million new cancer diagnoses are reported annually worldwide, which has become a major global public health problem (Siegel et al., 2024). Benign *in situ* tumor hyperplasia affects the normal physiological functions of some organs and systems; however, more than 90% of cancer mortality is caused by late malignant metastasis, recurrence, or discontinuous secondary lesions (Suhail et al., 2019; Ganesh and Massagué, 2021). Therefore, effective control or reversal of the growth and metastasis of tumor cells is of great significance in treating patients and is an important research topic.

Tumor invasion and metastasis are complex biological processes involving several oncogenes and tumor suppressor genes that regulate tumor growth and metastasis and several signaling pathways including Notch, JNK, Raf-MAPK, WNT, and Hippo (Lin et al., 2019; Zhang et al., 2019; Yang et al., 2022). The Notch gene was first identified approximately 110 years ago in *Drosophila*. Notch signaling is a highly conserved transduction pathway from flies to humans, consisting of Notch receptors, Notch ligands, transcription factors (TFs), and downstream target genes (Meurette and Mehlen, 2018; Zhou et al., 2022). Notch and Notch ligands are initially membrane proteins that interact with each other and undergo three-step proteolysis (S1-S3 cleavage), leading to the release of the Notch intracellular domain (NICD) (Conner, 2016; Huenniger et al., 2010). The NICD is transported into the nucleus and combines with ubiquitous TF CBF-1/suppressor of hairless/Lag1 (CSL, also termed recombination signal binding protein-J, RBPJ). CSL recruits Mastermind-like protein to activate the transcription of Notch target gene families Hairy/Enhancer of Split and Hairy/Enhancer of Split related to YRPW motif (Gomez-Lamarca et al., 2018; Bottoni et al., 2019). The Notch pathway is abnormal in a variety of malignant tumors including breast, non-small cell lung, and ovarian cancers (Shi et al., 2024; Leontovich et al., 2018), and is closely related to tumor occurrence, invasion, metastasis, the tumor microenvironment, and angiogenesis (Lu et al., 2016; Guo and Rafii, 2017; Meurette and Mehlen, 2018). In addition, Notch signaling may play an important role in maintaining cancer cell subsets with stem cell characteristics and resistance to chemotherapy, thereby enhancing cancer invasiveness (Pannuti et al., 2010). Several studies have suggested that the activation of Notch1 could promote epithelial-mesenchymal transition (EMT, a pivotal mechanism for cancer cells to acquire malignant properties) in hepatocellular carcinoma (Ang et al., 2023), which contributes to facilitating migration, invasion, and metastasis (Jin et al., 2019; Jing et al., 2019). Moreover, the Notch signal cascades play a central role in modulating tumor angiogenesis, which is regarded as an unusual feature of the tumor microenvironment, providing essential nutrients for the primary growth of tumor cells and the opportunity for malignant tumor cells to enter circulation, thus forming distant metastasis (Wang et al., 2020; Dudley and Griffioen, 2023).

*Taraxacum mongolicum* Hand. -Mazz., *Taraxacum sinicum* Kitag. (also termed *Taraxacum borealisinense* Kitam.), and other plants in the same genus and species of *Asteraceae* and *Taraxacum* F. H. Wigg. are perennial herbs with strong adaptability that grow in various soils. They are distributed worldwide and have extremely high edible and medicinal values (National Pharmacopoeia Commission, 2020; Zhang et al., 2022). As a traditional Chinese medicine, *Taraxaci* herba (dandelion, Chinese name: Pugongying) is used as a dry whole grass with roots. It has cholagogic, diuretic, antioxidant, anti-inflammatory, and liver protective characteristics and plays important roles in the treatment of various malignant tumors including breast, lung, and gastric cancers (He et al., 2019; Chen et al., 2021; Deng et al., 2021; Kang et al., 2021). Modern pharmacological research has shown that dandelion extract and several other effective ingredients have therapeutic effects on tumor invasion and metastasis. A previous study reported that dandelion ethanol extract inhibits the malignant invasion phenotype of MDA-MB-231 and MDA-MB-231 triple-negative breast cancer (TNBC) cells via alterations in the microenvironment of tumor-associated macrophages mediated by IL-10/STAT3/PD-L1 signaling (Deng et al., 2021). In addition, taraxerols inhibit the migration and invasion of TNBC MDA-MB-231 cells through the ERK/Slug signaling axis (Xia et al., 2023). The total flavonoids from *T. mongolicum* Hand. -Mazz. have an inhibitory effect on lung cancer, which may be due to the improvement of the host’s protective immune response via a milder tumor growth inhibitory effect than that of cyclophosphamide (Kang et al., 2021). Additionally, network pharmacological analysis and cell culture assays have been used to verify the anticancer activity of taraxerols in gastric cancer via multiple targets and pathways (Huo et al., 2022). Although these previous studies have confirmed the antitumor metastasis effects of dandelion, the dandelion parts and components that play central roles and the potential underlying molecular mechanisms have not yet been elucidated and require further investigation.

Due to factors such as harvesting origins, harvesting methods, and processing methods, the quality of available dandelion medicinal materials is not standardized, and common dandelion and alkaline-land dandelion products have not yet been distinguished. A saline-alkali environment has a significant impact on a plant’s secondary metabolites and nutrients; previous research found that dandelion strains grown in saline soil or under saltwater stress accumulate more active ingredients and are of better quality (Zhang et al., 2021; Meng et al., 2022; Wu et al., 2022). Therefore, the self-cultivated, high-quality, alkaline-soil dandelion strain Binpu-3 (*T. sinicum* Kitag.) was used in the current study. Dandelions were grown in saline-alkali soil and root and leaf extracts were prepared. A *Drosophila eyeful* tumor invasion model and a *ptc*>*scrib-IR* cell migration model were used to investigate the drug interventions. The results of this study suggest that Binpu-3 root extract (Binpu-3RE) inhibits tumor cell metastasis via regulation of Notch signaling activity and the expression of EMT-related factors β-integrin and matrix metalloproteinase-1 (MMP1). Moreover, Binpu-3RE may suppress growth, migration, and invasion in TNBC MDA-MB-231 cells. These findings provide a theoretical basis for the development and medicinal use of alkaline-soil dandelion resources and suggest potential drug targets for the clinical treatment of malignant tumors.

## 2 Materials and methods

### 2.1 Binpu-3 field planting

Binpu-3 and Handan (a wild dandelion strain collected in the non-saline area of Handan, Hebei Province, China) seeds were initially sown in a greenhouse and subsequently transplanted to the field once they developed 3–4 leaves. The seedlings were transplanted at a depth of 2–3 cm, with row spacing of 50 cm and ditch spacing of 30 cm. Throughout the cultivation period, an automatic negative-pressure drip irrigation system was used to maintain a soil matrix potential of −15 kPa. Urea was applied as topdressing at a rate of 15–22.5 g/m^2^ when the longest leaves reached 10–15 cm in length. A coastal, mildly saline-alkali soil with a salt content of 0.2%–0.3%, a soil bulk density of 1.4 g/cm^3^, a soil pH in the range of 7.8–8.5, and an organic matter content of 1.426% was used. We conducted a salt stress test in a salt tolerance identification pool with a soil salt content of 0.6% (Zhang et al., 2021).

### 2.2 Binpu-3 extract preparation

Whole Binpu-3 (or Handan) plants were collected and washed thoroughly. The aboveground parts (primarily leaves with a few flowers) and the underground parts (roots) were separated and dried at 60°C until the moisture content was <13%. The dried materials were ground and passed through a number 4 sieve to produce dry powders, which were stored as 100 g samples in gauze bags. The gauze bags were immersed in 1,000 mL of deionized water and boiled for 1 h. Subsequently, the residue was decocted again as described above. Lastly, the twice extracts were combined, centrifuged, filtered through a 0.45 μm membrane, and diluted to a final volume of 200 mL. The Binpu-3 leaf extract (Binpu-3LE) and Binpu-3RE stock solutions had final concentrations of 500 mg/mL.

### 2.3 High-performance liquid chromatography (HPLC) analysis

The contents of the extracts were detected using 0.75 g samples of dry powder incubated with 15 mL of 0.1% acid cellulase aqueous solution at 60°C for 30 min. Then, 15 mL of methanol was added and ultrasonicated over 30 min at 66°C. Lastly, the extract was centrifuged at 4,000 rpm for 10 min and filtered through a 0.45 µm membrane for high-performance liquid chromatography (HPLC), which was performed using a Shimadzu LC-20A system equipped with an Agilent Eclipse XDB-C18 column (4.6 × 250 mm, 5 μm; Agilent Technologies, Inc., city, CA, USA). The mobile phase flow rate was set at 1.0 mL/min. The mobile phase for phenolic acid (caftaric acid, chlorogenic acid, caffeic acid, and cichoric acid) detection was methanol (A) and 0.2% phosphoric acid (B) at a ratio of 35:65 (A:B). A wavelength of 327 nm was used to measure the different contents. The mobile phase used for the detection of flavonoids (quercetin, luteolin, and apigenin) was acetonitrile (C) and 0.5% phosphoric acid (D) at a ratio of 30:70 (C:D) and a measurement wavelength of 360 nm. The detection of triterpenoids (taraxasterol and taraxerol) was conducted using 0.01 mmol/L potassium dihydrogen phosphate as the mobile phase and measured at a wavelength of 210 nm.

### 2.4 Ultra performance liquid chromatography-high resolution mass spectrometry (UPLC-HRMS) analysis

A sample of the prepared Binpu-3RE solution was analyzed using a Vanquish ultra performance liquid chromatography (UHPLC) system (Thermo Fisher Scientific, Bremen, Germany) equipped with a ACQUITY UPLC HSS T3 column (2.1 mm × 100 mm, 1.8 µm) maintained at 35°C. The mobile phase consisted of 0.1% formic acid aqueous solution (A) and 0.1% formic acid acetonitrile solution (B); it was pumped at a flow rate of 0.3 mL/min. Gradient elution was performed according to Supplementary Table 2. Next, the Q-Exactive HFX mass spectrometer (Thermo Fisher Scientific, Germany) was coupled with the UHPLC system to collect the primary and secondary spectrograms of the sample in both positive and negative electrospray ionization (ESI) modes. The spray voltage was 3800V (ESI+)/3500V (ESI-), the sheath gas pressure was 45 arb, the auxiliary gas pressure was 20 arb, the ion transfer tube temperature was 320°C, and the atomization temperature was 350°C. The MS/MS spectrum was obtained using the top 10 MS1 ion, and the collision energies (CEs) were normalized by steps with energy levels of 20, 40, and 60. The scanning range of the first-order mass-to-charge ratio was 90–1300. Lastly, the format of the original data was converted, peak alignment, retention time correction, and peak extraction were carried out using XCMS software, and the chemical substances in the sample were identified by searching the local database of high-resolution mass spectrometry of traditional Chinese medicine.

### 2.5 *Drosophila* strains and genetics

Fly strains were stored at 25°C under 60% humidity in a 12 h light/dark cycle with a standard culture medium of 25 g sucrose, 30 g cornmeal, 5 g agar, 15 g yeast, 3 mL propionic acid, and 500 mL ultrapure water. The following *Drosophila* stocks were used: *w* ^*1118*^ (Bloomington *Drosophila* Stock Center, BL#3605), *UAS*-mGFP (BL#32197), *NRE*-GFP (BL#30728), and *UAS-scrib-IR* (Vienna *Drosophila* RNAi Center, VDRC#27424). The *ey*-GAL4, *ptc*-GAL4 *UAS*-GFP (*ptc*>GFP), and *eyeful* strains were gifts from Professor Lei Xue of Tongji University (Ferres-Marco et al., 2006; Wu et al., 2015; Wang et al., 2020). The *Delta*-LacZ and *Su(H)*-LacZ strains were gifts from Professor Xianjue Ma of Westlake University. The construction of the *ptc*>*scrib-IR* cell migration and *eyeful* tumor invasion models has been previously described (Wang et al., 2020). For all fly crossing experiments, healthy, unmated male and female parents were randomly assigned to different groups. We directly added the prepared Binpu-3LE or Binpu-3RE to the standard food to achieve final concentrations of 6.25, 12.50, 25.00, and 50.00 mg/mL. The corresponding offspring with the genotype *ptc>*GFP/+ or *ey*-GAL4/+ were kept in the control medium without any vehicle treatment as the control group. Offspring with *ptc>*GFP/*UAS-scrib-IR* or *eyeful*/+ were kept in the control or extract-supplemented medium as the model or drug-treated groups.

### 2.6 Food intake

Early *w* ^*1118*^ 3^rd^ instar larvae were washed with phosphate-buffered saline (PBS), starved for 2 h under adverse food conditions (PBS+0.8% agar), and transferred to fresh medium containing 0.5% Brilliant Blue FCF (Sangon Biotech, #A606319, China) for 20 min. Then, the larvae were washed with PBS and homogenized in 100 μL lysis buffer (PBS+0.1% Triton X-100) using a micro high-speed tissue homogenizer for 3 min. After centrifugation, 2 μL of the supernatant was measured with a spectrophotometer at a wavelength of 630 nm. A standard curve was generated using 2 mg of dye-containing food dissolved in 480 μL lysis buffer (Matsuda et al., 2015; Cao et al., 2022).

### 2.7 Real-time quantitative polymerase chain reaction

TRIzol (Sigma, #M3148) and a PureLink™ RNA Mini Kit (Life Technologies, #12183018A) were used to isolate the total RNA from the heads of 50 adult flies of each group. Real-time quantitative polymerase chain reaction (RT-qPCR) was performed as previously described (Wang et al., 2003). The relative amounts of transcripts were calculated using the comparative *C*_*T*_ method, and *ribosomal protein* 49 (*rp49*) was used as an internal control gene. The primer sequences that were used in this study are listed in Supplementary Table 1.

### 2.8 X-gal staining

The dissected larval tissues were fixed in Buffer A (50 mL 0.1% PBS containing Tween-20 (PBST) + 50 μL 1 mM MgCl_2_ + 1.5 mL 5M NaCl) containing 1% glutaraldehyde for 15 min at 25°C. Then, the tissues were rinsed once in Buffer A containing 3.3 mM K_3_Fe(CN)_6_ + 3.3 mM K_4_Fe(CN)_6_.3H_2_O, and incubated in Buffer A containing 3.3 mM K_3_Fe(CN)_6_ + 3.3 mM K_4_Fe(CN)_6_.3H_2_O + 0.2% 5-bromo-4-chloro-3 indolyl-β-D-galactopyranoside (X-gal, TIANGEN, #RT119) at 20°C–25°C (room temperature). The dyeing time of *Delta*-LacZ was 1 h and that of *Su(H)*-LacZ was 15 min. The tissues were then washed with Buffer A mounted with 100% glycerol.

### 2.9 Immunohistochemistry

Dissected larval tissues were fixed in 4% formaldehyde for 20 min at room temperature. After three washes with 0.3% (v/v) PBST, the tissues were stained with primary antibodies overnight at 4°C followed by staining with secondary antibodies for 4 h at room temperature. The following antibodies were used: mouse anti-MMP1 (1:200, Developmental Studies Hybridoma Bank (DSHB), #3A6B4, #14A3D2, #5H7B11), mouse anti-β-integrin (1:100, DSHB, CF.6G11), and goat anti-mouse-Cyanine3 (1:1000, Life Technologies, #A10521). Vectashield medium with 4′,6-diamidino-2-phenylindole (Vector Laboratories, #H-1500) was used for mounting. The fluorophores were excited and visualized using an inverted fluorescence system (Olympus, IX51).

### 2.10 Binpu-3RE-containing serum preparation

Thirty-two specific pathogen free (SPF) male SD rats (8 weeks, 180 ± 20 g) were obtained from the Experimental Animal Center of North China University of Science and Technology (Laboratory Animal Production license: SYXK (Ji) 2023–018). The animals were raised in an SPF room, on a light–dark cycle of 12 h each session, with an appropriate temperature (23 ± 2°C) and humidity (60 ± 5%). They were allowed free access to SPF-purified water. After 7 days of adaptive feeding, the rats were randomly divided into the blank control group, Binpu-3RE-low (Binpu-3RE-L, 2.73 g/kg), Binpu-3RE-medium (Binpu-3RE-M, 5.46 g/kg), and Binpu-3RE-high (Binpu-3RE-H, 10.92 g/kg) groups. Animals in all groups were given the corresponding dose of Binpu-3RE or the same volume of normal saline once a day. On the eighth day, they were anesthetized and blood was collected from the aorta, centrifuged at 3,000 rpm for 10 min to separate serum, inactivated in a water bath at 56°C for 30 min, and then filtered and sterilized using a needle filter. Lastly, the obtained rat control serum and Binpu-3RE-containing sera were stored at −20°C for future use.

### 2.11 Cell line and culture

Human breast cancer MDA-MB-231 cells obtained from FuHeng Biology (Shanghai, China, # FH0238) were incubated at 37°C in a water jacket incubator (Thermo Fisher Scientific, 311) under a 5% CO_2_ atmosphere with Dulbecco’s modified Eagle’s medium (DMEM) (EallBio, #C008) supplemented with 10 U/mL penicillin-streptomycin (NCM Biotech, China, #C100C5) and 10% fetal bovine serum (FBS) (Procell, China, #164210–50), 10% rat control serum, or rat Binpu-3RE-containing sera. Cells were passaged when they reached 80% confluence.

### 2.12 Cell viability assay (CCK-8)

MDA-MB-231 cells were passaged into 96-well plates at a density of 5 × 10^3^ cells/well. After 24 h, the cells were treated with DMEM supplemented with rat control serum (without any vehicle treatment) or Binpu-3RE-containing serum (Binpu-3RE-L, Binpu-3RE-M, Binpu-3RE-H) for 24 h, 48 h, and 72 h at 37°C under a 5% CO_2_ atmosphere. Subsequently, the cells were treated with a mixture of DMEM (90 μL) and cell counting kit (CCK)-8 solution (10 μL, Mei5 Biotech, China, #MF128-01), then incubated for 2 h. The absorbance was then measured at 450 nm using a plate reader (Tecan, M200PRD).

### 2.13 Wound healing assay

MDA-MB-231 cells were cultured in 6-well plates at a density of 5 × 10^4^ cells/well at 37°C under 5% CO_2_ saturation humidity until the degree of integration exceeded 90%. Next, a sterile 200 µL pipette tip was used to create a straight clean scratch wound across the center of each well, and marked “0 h”. Then, the cells were maintained in DMEM supplemented with rat control serum or Binpu-3RE-containing serum (Binpu-3RE-L, Binpu-3RE-M, Binpu-3RE-H) for 24 h, 48 h, and 72 h. Each wound was photographed at predetermined time points in the same position, and the migration was determined using ImageJ software. The following equation was used to determine the rate of cell migration: Cell migration rate (%) = (initial scratch area-final scratch area)/initial scratch area × 100%.

### 2.14 Transwell assay

The upper chamber of the transwell instrument was coated with diluted matrigel (1:8), and MDA-MB-231 human breast cancer cells were suspended in serum-free DMEM and seeded in the upper chamber at a density of 5.0 × 10^4^ cells/200 μL of cell suspension. To promote cell invasion, the lower chamber was filled with medium supplemented with 10% rat control serum or 10% Binpu-3RE-containing serum (Binpu-3RE-L, Binpu-3RE-M, Binpu-3RE-H) as a chemoattractant. Following this, the chambers were incubated at 37°C in a humid atmosphere containing 5% CO_2_ for 72 h. Next, non-invading cells on the upper surface were wiped off with cotton swabs, and the invading cells in the lower chamber were fixed with 1% crystal violet for 20 min, washed with PBS, and then stained and visualized using an inverted microscope. Lastly, the number of invading cells in each group was quantified using ImageJ software.

### 2.15 Western blotting

MDA-MB-231 cells were collected and extracted using radioimmunoprecipitation assay (RIPA) lysis buffer containing phosphatase inhibitors and 1% phenylmethanesulfonyl fluoride (PMSF). The total protein was quantified using the BCA quantification assay. Equal amounts of protein (10–30 µg) were separated by sodium dodecyl sulfate-polyacrylamide gel electrophoresis (SDS-PAGE), transferred to a polyvinylidene fluoride (PVDF) membrane, and subjected to the standard Western blot protocol, as previously described (Roth et al., 2018). The antibodies used were rabbit anti-Notch1 (1:1000, HUABIO, #SJ205), rabbit anti-Jagged1 (1:1000, HUABIO, #JF96-06), rabbit anti-Hes1 (1:1000, HUABIO, #SC06-21), rabbit anti-α-Tubulin (1:1000, Cell Signaling Technology, #2125S), and goat anti-rabbit IgG (H + L, HRP) (1:1000, Report, #S1002).

### 2.16 Data and statistics

GraphPad Prism (version 8.0) was used to create bar graphs of the study results. Comparison of data was performed using one-way analysis of variance (ANOVA) with Bonferroni’s multiple comparison test, unpaired t-test, chi-square test, Kruskal–Wallis one-way ANOVA test, or two-way ANOVA with Dunnett’s multiple comparison test. Statistical significance was set at *P <* 0.05, and the center values were considered the mean.

## 3 Results

### 3.1 Binpu-3 displays growth advantages and excellent quality in saline-alkali soil

To screen high-quality dandelion suitable for growth in saline-alkali areas, 22 dandelion strains were obtained via field collection or combined salt stress mutation breeding (Zhang et al., 2021). The Binpu-3 dandelion strain (initially termed Tanghai-6, according to the area and number at the time of collection) exhibited obvious advantages in terms of growth, fingerprint quality, *in vivo* antioxidant capacity, and anti-bacterial ability. Therefore, this strain was used to create a new variety (Hebei Province Drug Recognition, China, #2023–015), and was selected for use in the current study.

**FIGURE 1:**
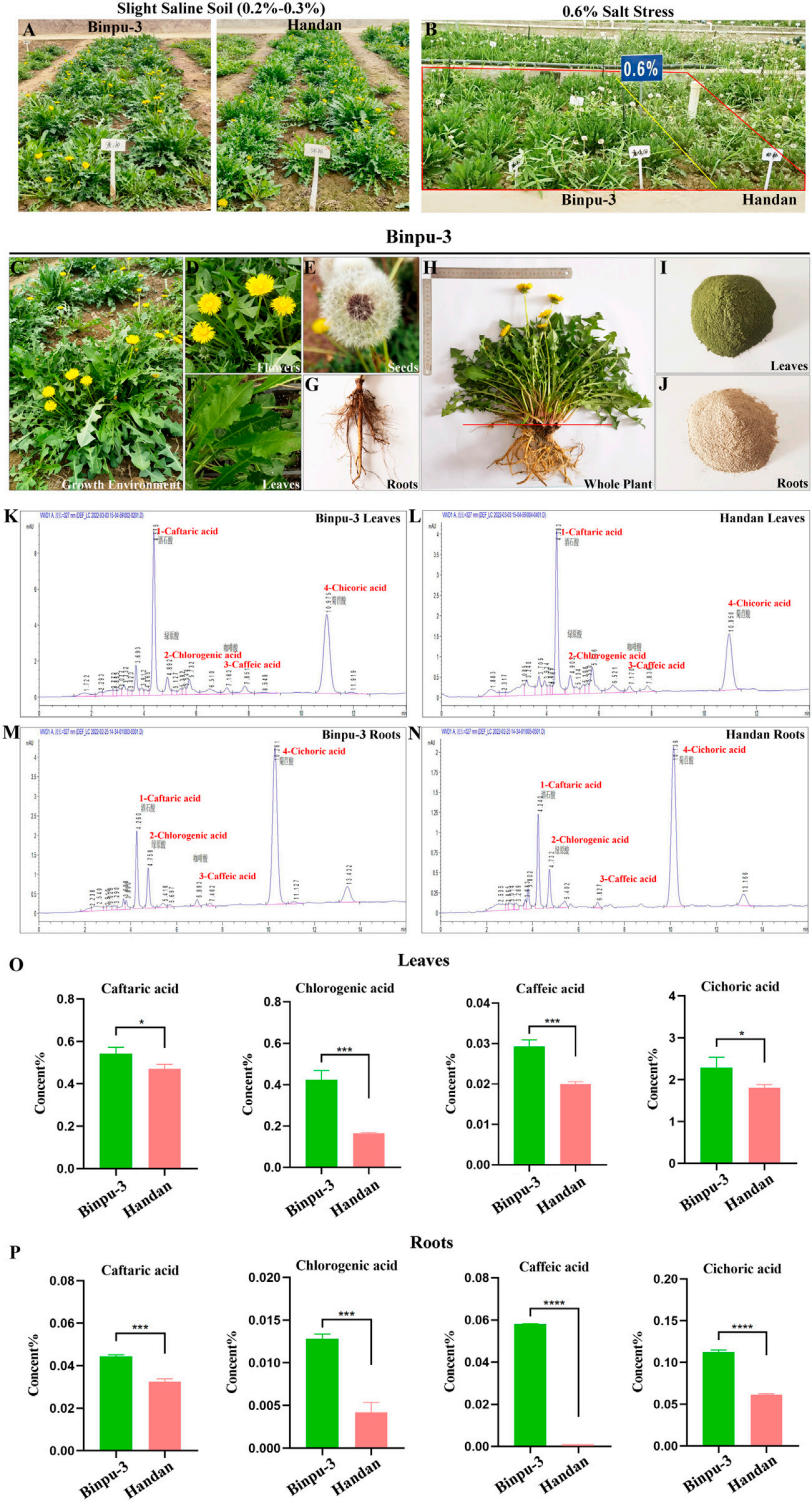
Cultivation and HPLC detection of Binpu-3 and Handan in alkaline soil. **(A, B)** Binpu-3 and Handan grown in a greenhouse with slight saline-alkali soil or in the 0.6% salt-tolerant identification pool. **(C–G)** Growth environment, flowers, seeds, leaves, and roots of dandelion Binpu-3. **(H–J)** Collected whole plant and dry powders of leaves and roots of Binpu-3. **(K–N)** Four phenolic acids (peaks 1–4 represent caftaric acid, chlorogenic acid, caffeic acid, and cichoric acid) were detected by HPLC in the leaves and roots of Binpu-3 or Handan. **(O, P)** Statistical analysis of the contents in indicated components (n = 3). Unpaired t-test was used to calculate the *P* value, ^∗∗∗∗^ *P* < 0.0001, ^∗∗∗^ *P* < 0.001, ^∗^ *P* < 0.05. Error bars indicate standard deviation.

Binpu-3 and Handan seeds were sown in a greenhouse and then transplanted into slight saline-alkali soil with a salt content of 0.2%–0.3%. Compared with the control Handan strain, Binpu-3 plants had a longer leaf length, longer leaf width, and greater height (Figure 1A). In addition, the growth mode of Binpu-3 was nearly vertical, with a narrow leaf shape, dark and hairless leaves, teeth at the crack edge, deep leaf cracks, and bright flowers under mild saline-alkali conditions (Figures 1C–G). Binpu-3 grew well in an environment of 0.6% salt stress (Figure 1B), indicating its strong salt resistance.

When the plant grew to maturity, whole plant samples of Binpu-3 and Handan were collected and separated into aboveground (mainly leaves, with a few flowers, hereafter referred to as leaves) and underground (roots) parts to make dry powders. The plant parts were cleaned, dried, crushed, and prepared into extracts for subsequent experiments (Figures 1H–J). Four effective components (phenolic acids) were detected using HPLC (Figures 1K–N; Supplementary Figure 1A). The caftaric, chlorogenic, caffeic, and cichoric acid levels in the leaves and roots of Binpu-3 were significantly higher than those in the leaves and roots of Handan (Figures 1O, P). Therefore, the Binpu-3 strain was considered of good quality and suitable for popularization and application in saline-alkali soil below moderate levels and in most regions of the world.

### 3.2 Binpu-3RE inhibits *eyeful* ocular tumor invasion in *Drosophila*

**FIGURE 2:**
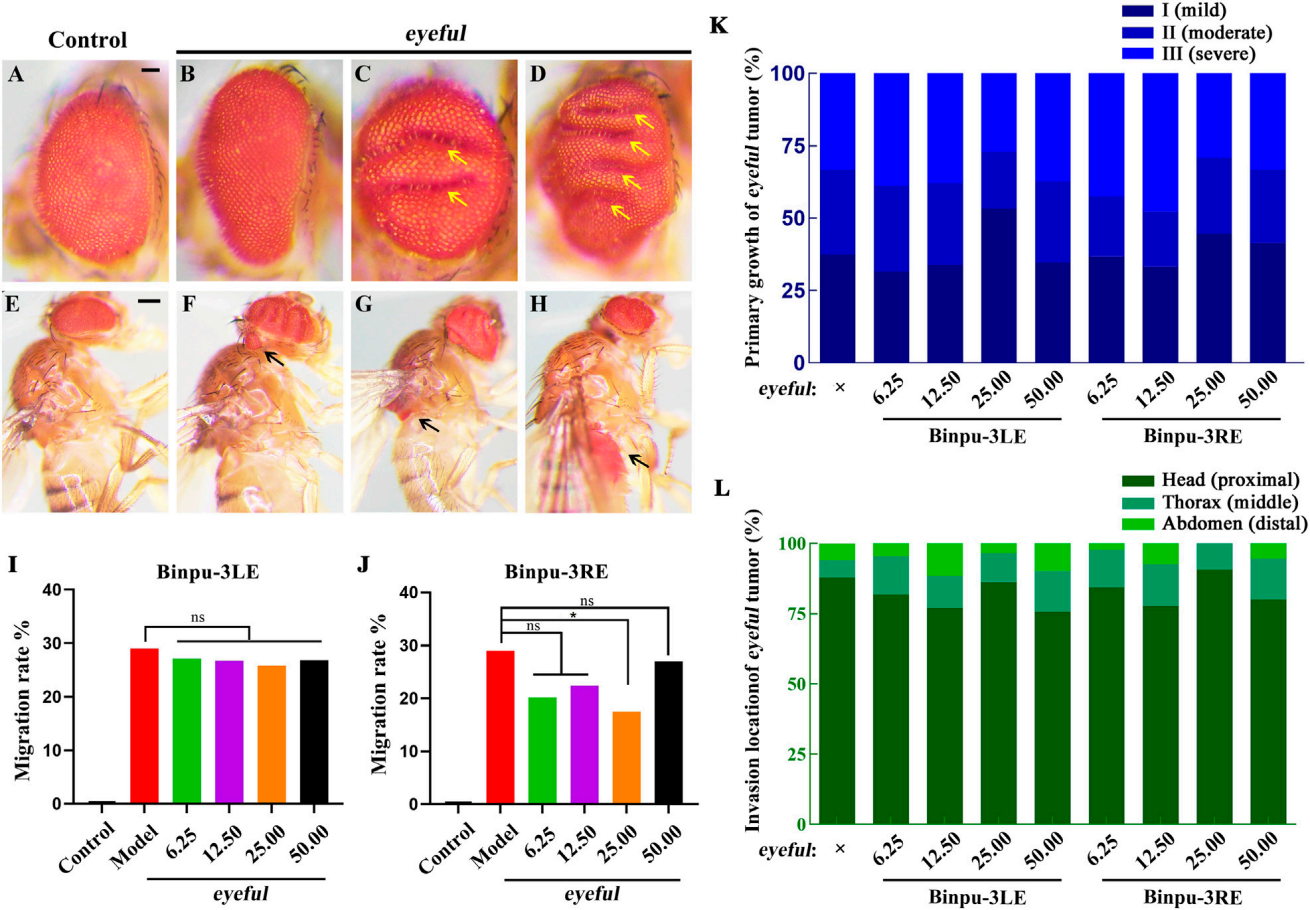
Binpu-3RE inhibits *eyeful* ocular tumor invasion in *Drosophila*. **(A–H)** Photographs showing the adult eye and body of the control (*ey*-GAL4) or *eyeful* flies. To evaluate the *in situ eyeful* tumor growth, *eyeful* flies without migrated tumors were divided into three subgroups, *viz.* I (no obvious fold, mild, **(B)**, II (1–2 folds, moderate, **(C)**, and III (≥3 folds, severe, **(D)** based on the number of folds (marked by yellow arrows) within the eye. To evaluate the invasion of *eyeful* tumor, *eyeful* flies with migrated tumors were divided into the head (proximal, **(F)**, thorax (middle, **(G)**, and abdomen (distal, **(H)** subgroups (the red eye tumor cells are indicated by black arrows) according to the invasion location of the ocular *eyeful* tumor. **(I, J)** Quantification of tumor cell migration percentage in indicated groups. The number of flies in each group with or without migrated tumor cells was recorded and calculated. The columns from left to right in i are: 1) Control (*ey*-GAL4, 0.00%, n = 123), 2) *eyeful* (28.99%, n = 169), 3) *eyeful* + Binpu-3LE 6.25 mg/mL (27.11%, n = 166), 4) *eyeful* + Binpu-3LE 12.50 mg/mL (26.73%, n = 101) 5), *eyeful* + Binpu-3LE 25.00 mg/mL (25.81%, n = 124), 6), and *eyeful* + Binpu-3LE 25.00 mg/mL (26.83%, n = 205). The columns from left to right in **(J)** are: 1) Control (*ey*-GAL4, 0.00%, n = 123), 2) *eyeful* (28.99%, n = 169), 3) *eyeful* + Binpu-3RE 6.25 mg/mL (20.18%, n = 109), 4) *eyeful* + Binpu-3RE 12.50 mg/mL (22.41%, n = 116) 5), *eyeful* + Binpu-3RE 25.00 mg/mL (17.47%, n = 166), 6), and *eyeful* + Binpu-3RE 25.00 mg/mL (26.97%, n = 152). A chi-squared test was applied to calculate the *P* value, ^∗^ *P* < 0.05, ns: no significant difference. **(K, L)** Stacked bar graphs of primary growth and invasion location in the *eyeful* tumor assay are shown (sample size details are shown in Supplementary Table 3). Data was analyzed using a Kruskal–Wallis one-way analysis of variance (ANOVA) test. Scale bar: 50 μm **(A–D)**, 200 μm **(E–H)**.

*Drosophila melanogaster* was used to construct an ocular tumor metastasis model, termed *eyeful* (Ferres-Marco et al., 2006; Wang et al., 2022). Compared with the control (Figures 2A, E), the eye tissues of the *eyeful* tumor model exhibited different degrees of tumor *in situ* proliferation (Figures 2B–D). Red-eye tumor cells migrated to other parts of the body including the head, thorax, and abdomen, at a migration rate of 28.99% (Figures 2F–I). Binpu-3LE and Binpu-3RE were added to the *Drosophila* standard culture medium at final concentrations of 6.25, 12.50, 25.00, and 50.00 mg/mL for a total of approximately 12 days from the embryonic stage until 2 or 3 days after emergence into adults. In detail, compared with the *eyeful* model, there was no significant difference in the composition ratio of primary growth and migration location of *Drosophila* eye tumors in the Binpu-3LE or Binpu-3RE treated groups (Figures 2K, L). Binpu-3RE treatment caused a reduction in the migration rate of eye tumor cells from 28.99% to 17.47% when applied at a concentration of 25.00 mg/mL (Figure 2J). However, Binpu-3LE had no obvious effect on tumor cell metastasis at any concentration (Figure 2I). Together, these data suggest that Binpu-3RE inhibits tumor cell metastasis at a suitable concentration in the *D. eyeful* model.

### 3.3 Analysis of effective components in the leaves and roots of Binpu-3

**FIGURE 3:**
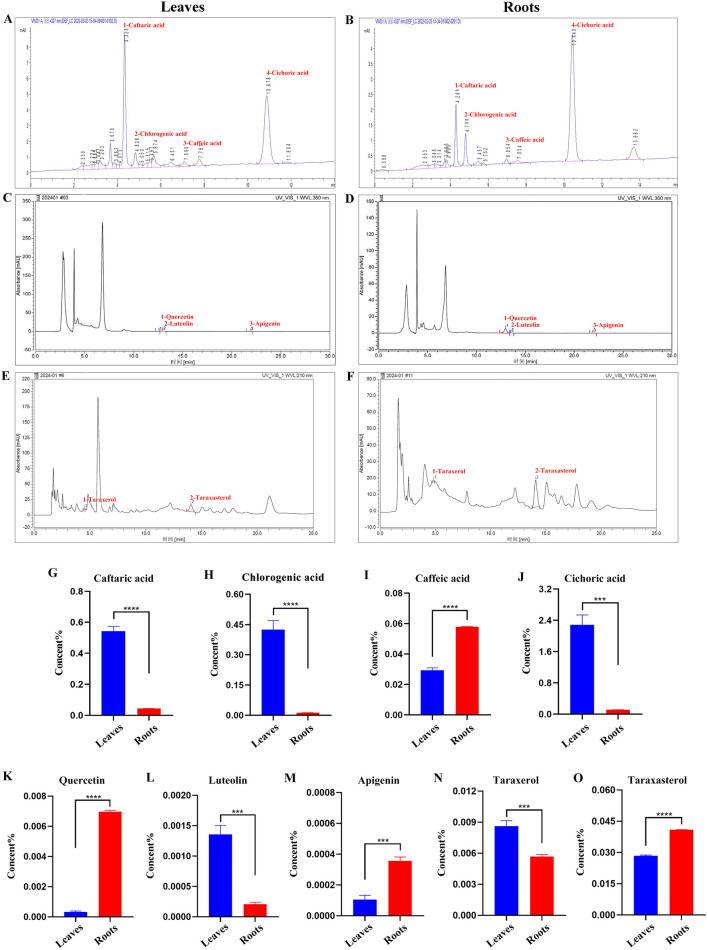
HPLC analysis of Binpu-3 leaves and roots. Four phenolic acids (peaks 1–4 represent caftaric acid, chlorogenic acid, caffeic acid, and cichoric acid) **(A, B)**, three flavonoids (peaks 1–3 represent quercetin, luteolin, and apigenin) **(C, D)**, and two triterpenoids (peaks 1–2 represent taraxerol and taraxasterol). **(E, F)** were identified in the Binpu-3 leaves and roots using HPLC. **(G–O)** Statistical analysis of the contents in indicated components (n = 3). Unpaired t-test was used to calculate the *P* value. ^∗∗∗∗^ *P* < 0.0001, ^∗∗∗^ *P* < 0.001. Error bars indicate standard deviation.

Why was Binpu-3RE capable of inhibiting *eyeful* tumor metastasis, whereas Binpu-3LE did not have an obvious inhibitory effect? To explore the potential reasons for this, we analyzed the contents of several active ingredients in Binpu-3 leaves and roots dry powder samples with HPLC (Supplementary Figure 1). The levels of four phenolic acids (caftaric acid, chlorogenic acid, caffeic acid, and cichoric acid) (Figures 3A, B), three flavonoids (quercetin, luteolin, and apigenin) (Figures 3C, D), and two triterpenes (taraxerol and taraxasterol) (Figures 3E, F) were assessed in Binpu-3 roots and Binpu-3 leaves. The caftaric acid, chlorogenic acid, cichoric acid, luteolin, and taraxerol levels were lower in leaves than in roots. However, the caffeic acid, quercetin, apigenin, and taraxasterol levels were significantly higher in Binpu-3 roots than those in Binpu-3 leaves (Figures 3G–O). These differences may account for the different effects of the two extracts.

### 3.4 UPLC-HRMS analysis of Binpu-3RE

**FIGURE 4:**
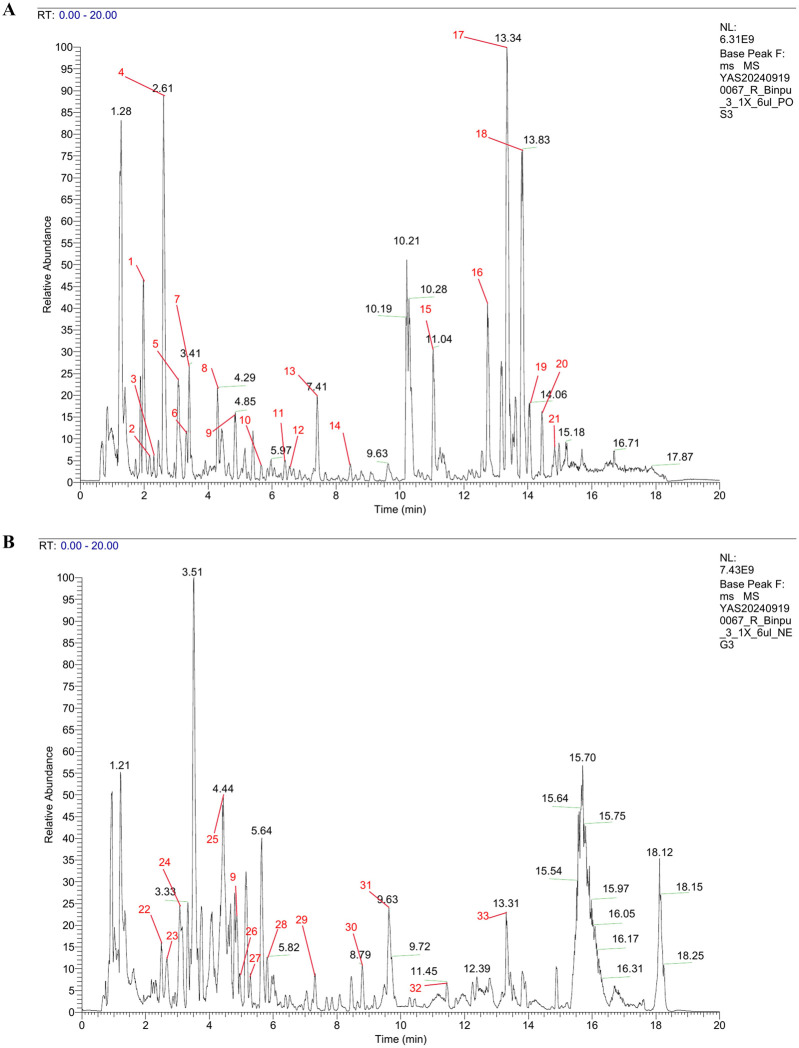
UPLC-HRMS analysis of Binpu-3RE. Base peak chromatograms (BPCs) of Binpu-3RE in in positive ion mode **(A)** and in negative ion mode **(B)**. The chromatographic peaks of 33 high-abundance components are marked by red numbers. The name, molecular formula, and content of these compounds are listed in Supplementary Table 4.

To comprehensively analyze the components in Binpu-3RE, we employed ultra-performance liquid chromatography combined high resolution mass spectrometry (UPLC-HRMS). The analysis data showed that 1349 components were detected in positive ion mode and 573 components were detected in negative ion mode, with a total of 1826 components being detected. Subsequently, we confirmed the peak shape of the chromatographic peaks with high abundance, checked the secondary chromatograms, and marked the chromatographic peak numbers of the positive and negative ion base peak chromatograms (BPCs) in numerical order (Figures 4A, B; red numbers). We discovered 33 major components with relatively high content, including pantothenate (0.1%), butein (0.53%), chlorogenate (0.78%), chicoric acid (1.96%), azelaic acid (0.23%), and [6]-gingerol (0.13%) (Figure 4; Supplementary Table 4).

### 3.5 Binpu-3RE suppresses *ptc*>*scrib-IR* induced cell migration

**FIGURE 5:**
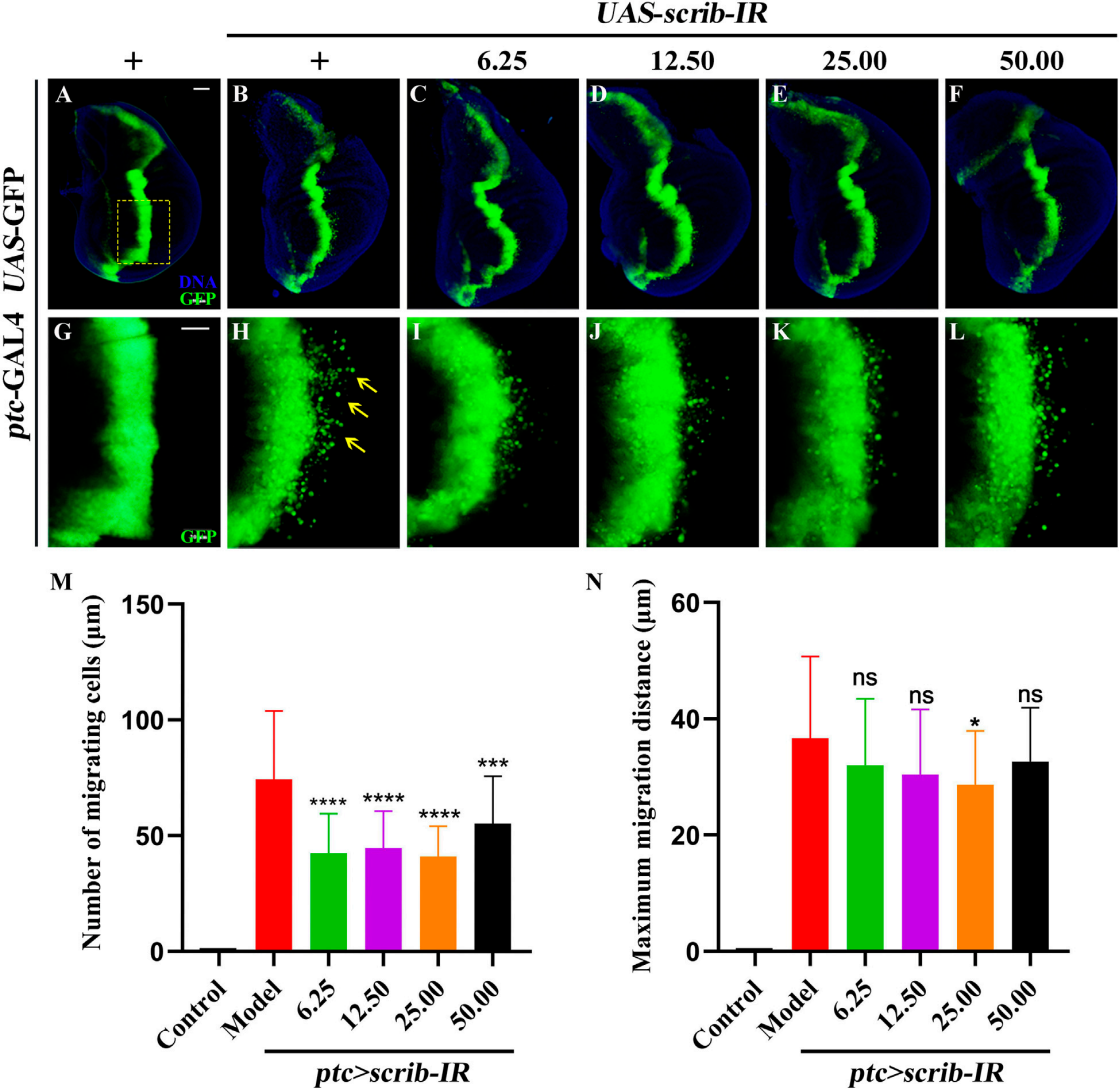
Binpu-3RE inhibits *ptc*>*scrib-IR* triggered cell migration. **(A–F)** Fluorescent microscopes of the 3rd instar larval wing imaginal discs are shown. **(G–L)** are high magnification of the yellow dotted boxed areas in **(A–F)**, respectively. The migrated GFP cells are indicated by the yellow arrows. Nuclei (DNA) are marked with DAPI (blue). Anterior is to the left and dorsal up. Statistical analysis of the migrating cell number **(M)** and maximum migration distance **(N)** for indicated groups (n = 30). One-way ANOVA analysis with Bonferroni’s multiple comparison test was used to calculate the *P* value. ^∗∗∗∗^ *P* < 0.0001, ^∗∗∗^ *P* < 0.001, ^∗^ *P* < 0.05, ns: no significant difference. Error bars indicate standard deviation. Scale bar: 50 µm **(A–L)**.

A well-recognized cell migration model of third-instar larval wing imaginal discs in *D. melanogaster* was used in this study (Zhang et al., 2019). Compared to the control (Figures 5A, G), RNAi-mediated downregulation of the cell polarity gene *scrib* (*UAS-scrib-IR*) driven by *ptc*-GAL4 along the anterior/posterior axis boundary area marked by GFP (*UAS*-GFP) (overall genotype abbreviated as *ptc*>*scrib-IR*) triggered a large number of cells to move to the rear within the wing pouch region, generating the cell migration phenotype (Figures 5B, H, M, N). The number of migrating cells was significantly reduced after treatment with 6.25, 12.50, 25.00, and 50.00 mg/mL Binpu-3RE (Figures 5C–F, I–M), with inhibition of the maximum migration distance after treatment with 25.00 mg/mL Binpu-3RE (Figure 5N). Therefore, this concentration was used for mechanistic research. Based on the above results, we concluded that Binpu-3RE could inhibit the *ptc*>*scrib-IR* triggered cell migration in *Drosophila* at an appropriate concentration, and 25.00 mg/mL was selected as the best dosage to carry out the follow-up mechanistic research.

Additionally, the experimental study of the corresponding *Drosophila* food intake was performed to verify whether the administration medium at each concentration affected feeding rate. The *Drosophila* fed with Binpu-3RE medium containing concentrations of 6.25, 12.50, 25.00, and 50.00 mg/mL, after starvation for 2 h, were adequately fed for 20 min in contrast with those fed with ordinary culture medium. No significant difference (ns, *P* > 0.05) was observed, excluding the possibility that changes in feeding rates may interfere with the tumor invasion and cell migration phenotype, and could be used for subsequent experiments (Supplementary Figure 2).

### 3.6 Binpu-3RE downregulates mRNA levels of key factors in the Notch pathway

**FIGURE 6:**
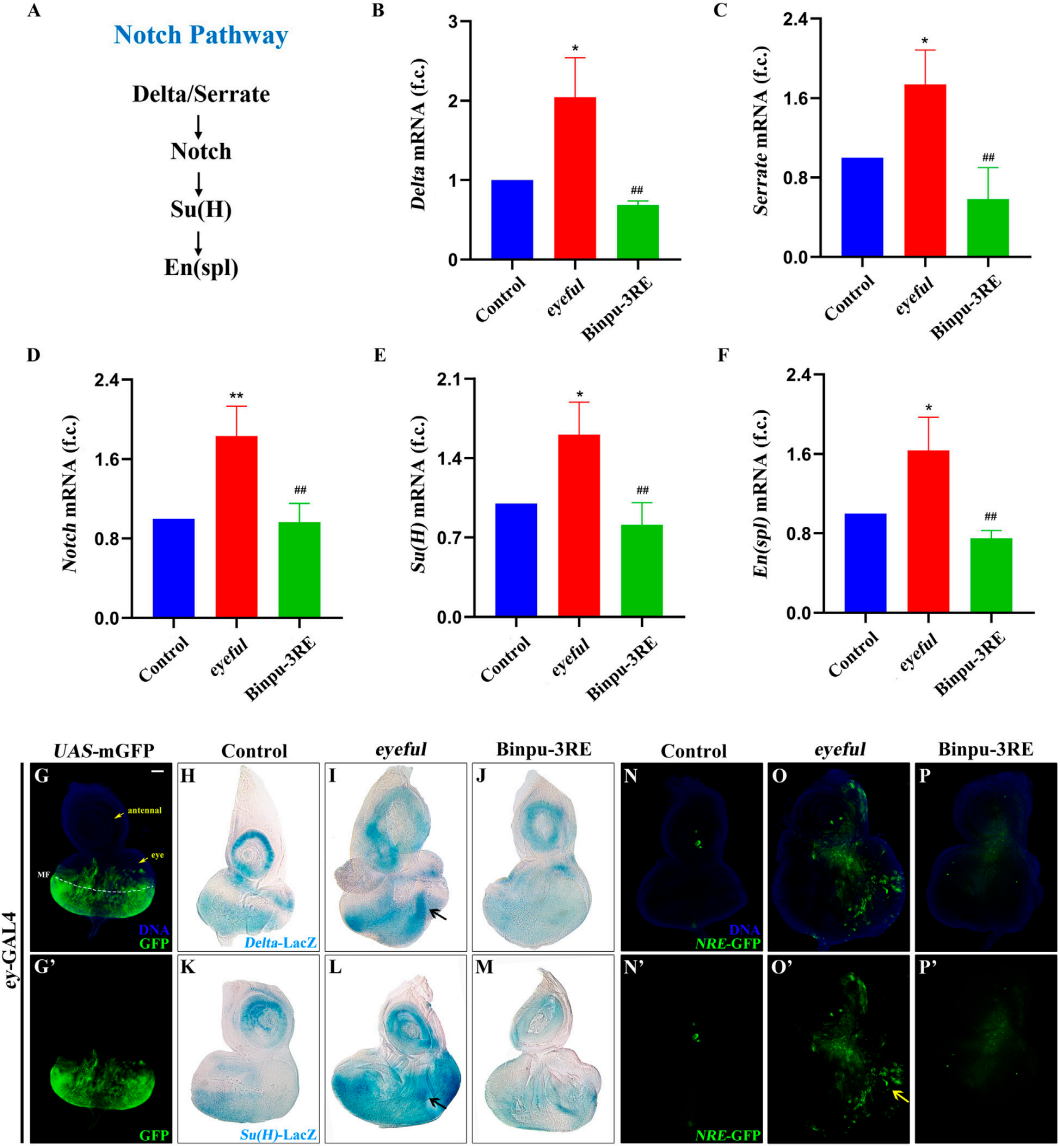
Binpu-3RE suppresses Notch signal activity. **(A)** Diagram for the key components of Notch signaling. **(B–F)** mRNA levels of *Delta*, *Serrate*, *Notch*, *SuH*, and *En(spl)* as measured by RT-qPCR (n = 3). One-way ANOVA with Bonferroni’s multiple comparison test was employed to compute the *P* value. ^∗∗^ or ^∗^ represents *eyeful* group compared with control group, *P* < 0.01 or *P* < 0.05; ^##^ represents Binpu-3RE group compared with *eyeful* group, *P* < 0.01. Error bars indicate standard deviation. **(G, N–P)** Merged fluorescent microscopes of the third instar larval eye-antennal imaginal discs. The antennal disc, eye disc, and morphogenetic furrow (MF) are respectively indicated by the yellow arrow or white dotted line in **(A)**. The Notch signaling activity was monitored via the reporter *NRE*-GFP in **(N–P)**. The yellow arrow indicates the increased expression of *NRE*-GFP in the *eyeful* group. Nuclei (DNA) are labelled with DAPI (blue). **(H–M)** X-gal staining of *Delta*-LacZ and *SuH*-LacZ reporters in eye-antennal discs. Black arrow indicates the increased expression level of *Delta*-LacZ or *SuH*-LacZ in the *eyeful* model. Scale bar: 50 μm **(G–P)**.

Due to the ectopic expression of Delta, a ligand for the Notch pathway, in the *eyeful* tumor model and the key role of Notch signal in tumor metastasis, the mRNA expression levels of key factors in the Notch signal pathway were detected using RT-qPCR including *Delta* (FlyBase ID: FBgn0000463), *Serrate* (FBgn0004197), *Notch* (FBgn0004647), *Suppressor of Hairless* (*Su(H)*, FBgn0004837), and *Enhancer of split* (*En(spl)*, FBgn0000591) (Figure 6A). The mRNA levels of these genes were significantly enhanced in the *D. eyeful* tumor metastasis model compared to those in the control model. After treatment with 25.00 mg/mL Binpu-3RE, the enhanced mRNA expressions of these genes’ mRNA were impeded (Figures 6B–F), indicating that Binpu-3RE could downregulate the transcription levels of *Delta*, *Serrate*, *Notch*, *Su(H)*, and *En(spl)* in the Notch pathway.

### 3.7 Binpu-3RE suppresses Notch signal activity *in vivo*

The reporter markers *Delta*-LacZ and *Su(H)*-LacZ were used to detect the expression of *Delta* and *Su(H) in vivo* using an X-gal staining assay. The eyeless (ey) protein is expressed in all cells of the eye discs at the second-instar stage and is expressed anterior to the morphogenetic furrow (MF) but not in the anterior-most part of the eye discs (where it contacts the antennal disc) at the third-instar larval stage (Bessa et al., 2002). The enhanced membrane GFP (mGFP)-labeled expression region of the *ey*-GAL4 driver was mainly located near the MF in the third instar larval eye-antennal imaginal discs (Figures 6G, G’). Compared with the *ey*-GAL4 control (Figures 6H, K), the expression of *Delta*-LacZ or *Su(H)*-LacZ was significantly upregulated both in the antennal and eye discs of the *eyeful* tumor model at the third instar larval stage, and an increased volume was observed (Figures 6I, L). Therefore, the transcription of *Delta* or *Su(H)* was enhanced in the *eyeful* tumor in both a cell-autonomous and non-autonomous manner. After treatment with 25.00 mg/mL Bingpu-3RE, the increased *Delta*-LacZ and *Su(H)*-LacZ levels were inhibited (Figures 6J, M), implying that Binpu-3RE restrains the mRNA levels of *Delta* and *Su(H) in vivo*.

A transgenic fly line for the *NRE*-GFP construct, in which enhanced GFP expression marked cells with active *Su*(H)-dependent Notch signaling, was used to visualize canonical Notch signaling activity in *D. eyeful* tumors *in vivo* (Saj et al., 2010; Beira et al., 2018). The expression of *NRE*-GFP was low in the control group (Figures 6N, N’) and strongly promoted in the third instar larval eye-antennal imaginal discs (Figures 6O, O’). However, this increased expression and enlargement of the eye-antennal discs was inhibited after treatment with 25.00 mg/mL Binpu-3RE (Figures 6P, P’) as well as the enlarged size of the eye-antennal discs. Together, these data suggest that Binpu-3RE can effectively inhibit the activity of the Notch signaling pathway *in vivo*.

### 3.8 Binpu-3RE inhibits the expression of β-integrin and MMP1

**FIGURE 7:**
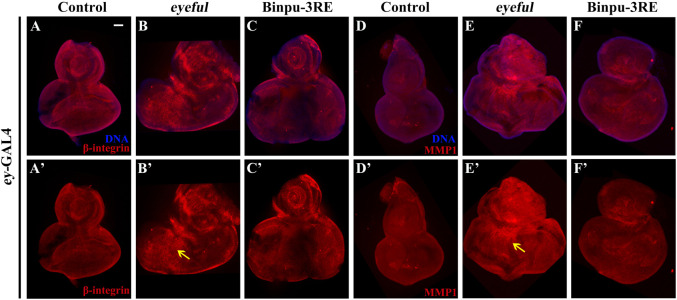
Binpu-3RE inhibits the expression of β-integrin and MMP1. Merged fluorescence microscopes showing the third instar larval eye-antennal discs stained with anti-β-integrin **(A–C)** and anti-MMP1 antibody **(D–F)**. The individual channels detect only β-integrin (red, **A’–C’**), and only MMP1 staining (red, **D’–F’**). The black arrow indicates the increased expression level of β-integrin or MMP1 in the *eyeful* model. Nuclei (DNA) are labeled with DAPI (blue). Scale bar: 50 μm **(A–F)**.

Based on the close relationship between Notch signal and EMT and their central roles in tumor cell migration and metastasis (Espinoza et al., 2013; Gonzalez and Medici, 2014), the expressions of β-integrin and MMP1 were measured using immunohistochemistry staining to identify potential mechanisms. Compared to the control (Figures 7A, A’, D, D’), protein expression levels of β-integrin and MMP1 were upregulated in the *eyeful* tumor model, though the upregulation was inhibited after treatment with 25.00 mg/mL Binpu-3RE (Figures 7B, B’, C, C’, E, E’, F, F’). Hence, we speculated that Binpu-3RE may interfere with the EMT process by inhibiting the expression of β-integrin and MMP1, thereby regulating tumor metastasis.

### 3.9 Binpu-3RE reduces viability, migration, invasion, and Notch signaling of breast cancer cells

**FIGURE 8:**
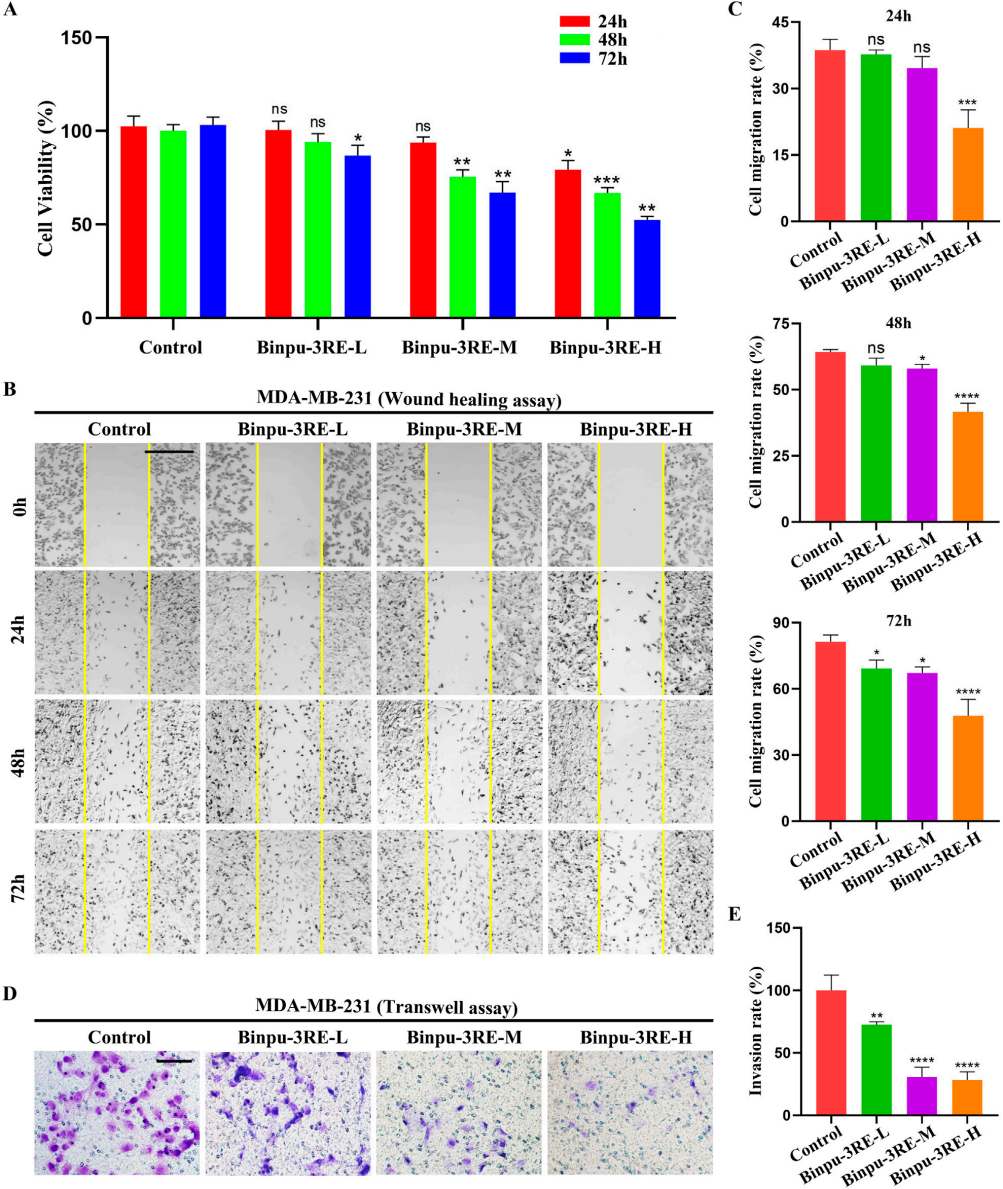
Binpu-3RE reduces viability, migration, and invasion of breast cancer cells. **(A)** The effects of Binpu-3RE-containing serum (Binpu-3RE-L, Binpu-3RE-M, Binpu-3RE-H) on the viability of MDA-MB-231 cells for 24 h, 48 h, and 72 h. Two-way ANOVA with Dunnett’s multiple comparison test was used to compute the *P* value **(B)** Cell migration using the wound healing assay. Images were taken at 0 h, 24 h, 48 h, and 72 h. The boundaries of the scratched wounds are outlined by yellow lines. **(D)** Cell invasion using the transwell assay. **(C)** Statistical analysis of cell migration rate **(C)** and invasion rate **(E)** for indicated groups (n = 3). One-way ANOVA analysis with Bonferroni’s multiple comparison test was used to calculate the *P* value. ^∗∗∗∗^ *P* < 0.0001, ^∗∗∗^ *P* < 0.001, ^∗∗^ *P* < 0.01, ^∗^ *P* < 0.05, ns: no significant difference. Error bars indicate standard deviation. Scale bar: 500 μm **(B)**, 100 μm **(D)**.

To evaluate the effect of Binpu-3RE on human breast cancer cells, we prepared rat control serum and Binpu-3RE-containing serum (Binpu-3RE-L, Binpu-3RE-M, Binpu-3RE-H) for use in treating MDA-MB-231 TNBC cells. Compared with the control (treated rat control serum), the cell viability of MDA-MB-231 cells was obviously impeded by Binpu-3RE-H at 24 h, by Binpu-3RE-M and Binpu-3RE-H at 48 h, and by all Binpu-3RE-containing sera at 72 h (Figure 8A), as assessed using the CCK-8 assay. Next, we employed the *in vitro* wound healing and transwell assays to assess the cell migration and cell invasion capabilities of MDA-MB-231 cells, respectively. Following exposure of Binpu-3RE at different doses, the cell migration and invasion rates were both largely suppressed in the MDA-MB-231 cell line (Figures 8B–E).

**FIGURE 9:**
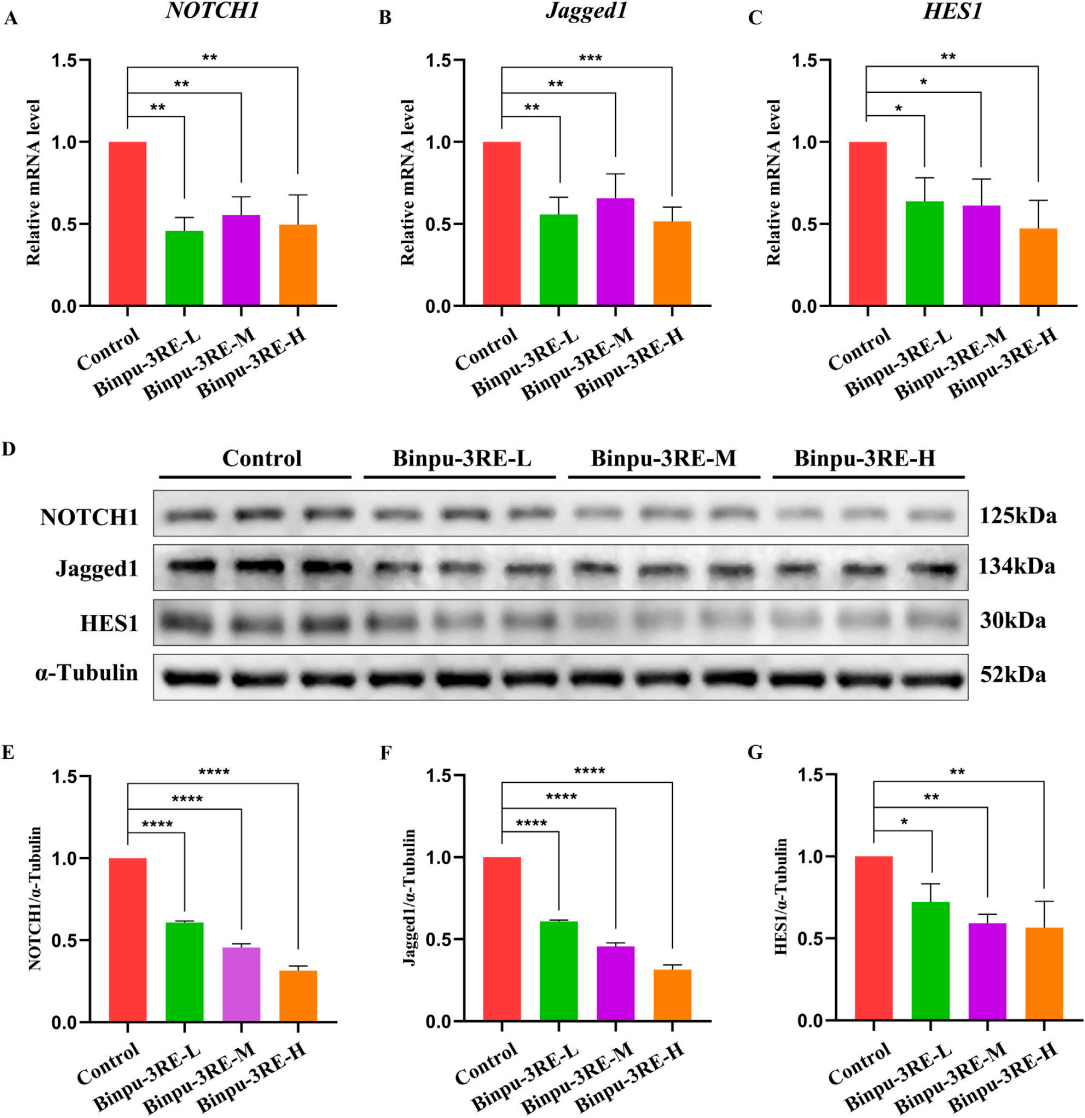
Binpu-3RE decreases Notch signaling in breast cancer cells. **(A–C)** mRNA levels of *NOTCH1*, *Jagged1*, and *HES1* in MDA-MB-231 cells as measured using RT-qPCR (n = 3). **(D)** Western blotting analysis of MDA-MB-231 cells for NOTCH1, Jagged1, HES1, and α-tubulin. α-Tubulin was used as an internal control. **(E–G)** Statistical analysis of the NOTCH1/α-tubulin, Jagged1/α-tubulin, and HES1/α-tubulin relative levels from three independent experiments are shown in **(D)** (n = 3). One-way ANOVA with Bonferroni’s multiple comparison test was employed to compute the *P* value. ^∗∗∗∗^ *P* < 0.0001, ^∗∗∗^ *P* < 0.001, ^∗∗^ *P* < 0.01, ^∗^ *P* < 0.05. Error bars indicate standard deviation.

As the Notch signaling pathway is highly conserved from fly to human, we further detected the level of Notch signaling in MDA-MB-231 breast cancer cells. As hypothesized, mRNA and protein expression levels of the key factors in the Notch signaling pathway in mammals, *viz.* Notch1, Jagged1, and HES1 were distinctively reduced by Binpu-3RE treatment (Figure 9). Together, these data imply that the positive effect of Binpu-3RE on tumor cell migration and invasion is through inhibiting Notch signaling with evolutionary conservatism.

## 4 Discussion

*Drosophila melanogaster*, with its small size, large number of offspring, short life cycle, non-redundant genetic background, low cost, convenient maintenance, and lack of ethical concerns, has served as an important model organism for studying human diseases (Ugur et al., 2016). More than 70% of human disease genes have orthologs in the fruit fly. Many genes related to tumorigenesis and metastasis, as well as fundamental biochemical pathways and signal transduction pathways, are highly conserved from *Drosophila* to mammals (Chatterjee and Deng, 2019; Hales et al., 2015). Additionally, the availability of numerous phenotypic markers that facilitate genetic manipulation has made *Drosophila* one of the effective models for studying the molecular regulatory networks of human tumorigenesis, cell migration, and apoptosis, as well as for large-scale screening of traditional Chinese medicine formulas, single herbs, and monomeric compounds, and investigating their targets of action (Wu et al., 2015; Wu et al., 2021; Wang et al., 2022).

As a heat-clearing and detoxicating Chinese herbal medicine, dandelion is mostly used as a whole herb (Zhang et al., 2022). This study’s findings showed that the levels of the four phenolic acids were higher in the Binpu-3 strain than in the Handan strain (Figures 1K–P). Caffeic and cichoric acids are quality markers of *T. mongolicum* Hand. -Mazz*.* and *T. sinicum* Kitag., confirming the high quality of the Binpu-3 strain used in this study.

In the *D. eyeful* ocular tumor assay, Binpu-3RE lowered the migration rate of the tumor cells at a suitable concentration, though Binpu-3LE had no obvious inhibitory effect. Which components of Binpu-3 root play the anti-tumor potential roles in metastasis? To answer this question, we first checked the contents of several widely studied effective medicinal components in dry powders of Binpu-3 leaves and roots using HPLC, and found that the levels of caffeic acid, quercetin, apigenin, and taraxasterols were significantly higher in Binpu-3 roots than in Binpu-3 leaves (Figure 3). Caffeic acid is a powerful anti-tumor agent and a prominent compound in studies regarding the development of new anti-cancer therapies (Bastidas et al., 2022). Quercetin and apigenin are flavonoids with antitumor effects, which may account for the antitumor metastatic effects of Binpu-3RE (Reyes-Farias and Carrasco-Pozo, 2019; Imran et al., 2020). Triterpenoids, especially taraxasterols, have a wide range of anti-tumor effects, achieved via the induction of cell apoptosis, inhibition of cell proliferation, and tumor angiogenesis (Jiao et al., 2022). Binpu-3RE and Binpu-3LE exhibited significant differences in overall efficacy in the current study. Caffeic acid, quercetin, apigenin, and taraxasterols, which are abundant in the roots, may exist in more optimized proportions and act synergistically on tumor cells via various mechanisms to inhibit metastasis and invasion.

Next, we carried out UPLC-HRMS analysis to comprehensively test the components of Binpu-3RE, and identified 33 compounds with high abundance, including pantothenate (0.1%), butein (0.53%), chlorogenate (0.78%), chicoric acid (1.96%), azelaic acid (0.23%), and [6]-gingerol (0.13%) (Figure 4; Supplementary Table 4). Among them, pantothenate (vitamin B_5_), a component of coenzyme A, inhibits the growth of tumor cells and enhances chemotherapy sensitivity by regulating the energy metabolism of tumor cells and exerting antioxidant effects (Kreuzaler et al., 2023). Butein inhibits tumor cell proliferation, induces apoptosis, and impedes angiogenesis (Dong et al., 2022). Chlorogenate can inhibit the inflammatory response and the process of cellular senescence. In addition, it can precisely regulate the cell signaling pathways and effectively suppress the EMT process (Nwafor et al., 2022). Chicoric acid exhibits anti-inflammatory properties, resists oxidative stress, and induces the differentiation and autophagy of tumor cells (Sun et al., 2019). Although the underlying mechanisms require further research, together, these findings provide a scientific basis for the further development and utilization of *T. sinicum* Kitag.

The *eyeful* tumor invasion model was established by crossing corresponding transgenic fly strains using a robust GAL4/UAS dual expression system (Brand and Perrimon, 1993; Ferres-Marco et al., 2006; Zhang et al., 2019) to ectopically express Delta (a Notch pathway ligand) and downregulate the epigenetic silencing factors *pipsqueak* (*psq*) and *longitudinals lacking* (*lola*). The results of the current study suggest that Binpu-3RE decreased the transcription level of the Notch pathway core components *Delta*, *Serrate*, *Notch*, *Su(H)*, and *En(spl)* and the activity of *in vivo* Notch signal (Figures 5, 6). The Notch pathway plays important roles in cell fate, adhesion, differentiation, apoptosis, and proliferation and is closely related to EMT (D’Assoro et al., 2022). *Su(H)* acts as a transcriptional activator that triggers the transcription of downstream target genes, which is crucial for the functional activation of Notch signaling (Morel and Schweisguth, 2000). In addition, decreased expression of *Su(H)* can significantly inhibit cell growth via suppression of its target genes CDK2, CDK4, CyclinD1, and Bcl-2, and upregulation of P21 and P27 to induce cell cycle arrest in prostate cancer cells (Xue et al., 2015). *En(spl)* is a target gene of the Notch signaling pathway, and its overexpression can trigger cell proliferation (Zacharioudaki et al., 2012). It is regarded a momentous medium for tumor *in situ* proliferation and malignant development, and its downregulation may slow the growth of xenograft tumor tissues (Voutyraki et al., 2022).

In *Drosophila* assays, Binpu-3LE or Binpu-3RE was administered into the standard fly food at concentrations of 6.25,12.50, 25.00 or 50.00 mg/mL, which were deduced from the China Pharmacopoeia (2020 Edition), several related previous studies (Choi et al., 2010; Deshpande et al., 2014; Slack et al., 2015), and our preliminary experiment. Following this, 25.00 mg/mL was selected as the dosage for Binpu-3RE to exert the best anti-tumor invasion effect, which is roughly equivalent to a human treatment dosage of 50 g (Binpu-3 root dry powder)/day. However, it should be noted that considering the multiple factors influencing the effect of drugs in different animal species, including the chemical properties of the drugs, their bioavailability, metabolic pathways, and excretion modes, this dosage (50 g/day) is only intended as a reference and cannot accurately reflect the actual optimal dosage of Binpu-3 root in humans. Besides, Qu *et al.* found that dandelion ameliorates doxorubicin-induced cardiotoxicity and attenuates its cytotoxicity in TNBC via activating the P-glycoprotein in heart and tumor tissues (Qu et al., 2022). Dandelion seed extract has been confirmed to affect tumor progression and improve the sensitivity of cisplatin in esophageal squamous cell carcinoma (ESCC) (Li et al., 2022). A previous study suggested that the Sophora alopecuroide-Taraxacum decoction (STD, a traditional Chinese herbal formulation) induces ferroptosis and modulates the tumor immune microenvironment to combat non-small cell lung cancer (NSCLC) (Ouyang et al., 2024). In consideration of the above literature, Binpu-3RE might become a viable alternative treatment or synergistic agent for use with chemotherapeutic drugs (doxorubicin, cisplatin, etc.) in clinical practice for patients with TNBC, ESCC, NSCLC, or other malignant tumors.

As dandelion displays certain therapeutic effects against breast cancer in clinical settings, this work also determined the effect of Binpu-3RE on the TNBC MDA-MB-231 cell line. Consistently, Binpu-3 significantly inhibited growth, migration, and invasion of MDA-MB-231 cells, and downregulated the expression of Notch signaling pathway key factors Notch1, Jagged1, and HES1 (Figures 8, 9), further confirming its ability to inhibit metastasis, which is clinically relevant. Because we only tested a single breast cancer cell line, more human cancer lines or *in vivo* mammalian models are needed for further validation of the efficacy of Binpu-3RE. In addition, considering adverse reactions or side effects, our subsequent work will also focus on the potential long-term effects of Binpu-3RE intervention for a comprehensive evaluation. Moreover, tumor invasion is a complex disease with other multiple contributing factors and pathways, including oncogenes, tumor suppressors, autophagy, oxidative stress, JNK signaling, Raf-MAPK signaling, WNT signaling, and Hippo signaling. Therefore, we plan to explore the influence of Binpu-3RE on the above related signal pathways or factors in the future.

## 5 Conclusion

In summary, our data provided solid evidences demonstrating that Binpu-3RE (the root extract of a *T. sinicum* Kitag. strain) significantly inhibits metastasis of tumor cells by regulating the Notch signaling pathway and the expression of EMT-associated factors β-integrin and MMP1 in *Drosophila*, as well as growth, migration, and invasion of MDA-MB-231 cells. These findings provide new insights and experimental evidence for the clinical treatment of triple negative breast cancer and other related malignant diseases.

## Data Availability

The original contributions presented in the study are included in the article/Supplementary Material, further inquiries can be directed to the corresponding authors.
