# Supplementary material for: Taraxacum sinicum Kitag. (Binpu-3) root extract inhibits tumor invasion via Notch signaling in Drosophila and human breast cancer MDA-MB-231 cells
Source: Front Pharmacol. 2025 Mar 13;16:1494545. doi: 10.3389/fphar.2025.1494545 (PMC11947688; doi:10.3389/fphar.2025.1494545)

Supplementary Materials

*Taraxacum sinicum* Kitag. (Binpu-3) root extract inhibits tumor invasion via Notch signaling in *Drosophila* and human breast cancer MDA-MB-231 cells

**Jiawei Wu^1#^, Jianbo Zhang^1#^, Wanyu Shu^1^, Wei Feng^2^, Ran Meng^2^, Lingyu Kong^3^, Huijuan Cao^1^, Chunhua Jiang, Sitong Wang^1^, Fanwu Wu^1^, Chenxi Wu^1,4^* and Xiuping Wang^2^***

^1^ Hebei Key Laboratory of Integrated Traditional Chinese and Western Medicine for Diabetes and Its Complications, College of Traditional Chinese Medicine, North China University of Science and Technology, 21 Bohai Road, Tangshan 063210, China

^2^ Institute of Coastal Agriculture, Hebei Academy of Agriculture and Forestry Sciences, 63 Binhai Road, Tangshan 063299, China

^3^ Oncology of Chinese and Western Medicine, North China University of Science and Technology Affiliated Hospital, 73 Jianshe Road, Tangshan 063000, China

^4^ Hebei Key Laboratory of Medical Engineering and Integrated Utilization of Saline alkali Land, Hebei Administration of TCM Key Laboratory of Quality Control of Salt alkali Resistant TCM, 21 Bohai Road, Tangshan 063210, China

^#^ These authors contributed equally to this work.

^*^Correspondence should be addressed to [chenxi.wu@ncst.edu.cn](mailto:chenxi.wu@ncst.edu.cn), [bhswxp@163.com](mailto:bhswxp@163.com)

**Supplementary Materials**

**Table S1. The primer sequences**

| **Gene name** | **Sequences** |
| --- | --- |
| *rp49* | Sense: 5’- CTTCATCCGCCACCAGTC -3’  Antisense: 5’- GCACCAGGAACTTCTTGAATC -3’ |
| *Delta* | Sense: 5’-AAACCGAAGACAACCAGACCTT-3’  Antisense: 5’-AGCTGCACAGTTTGTTTCTAGC-3’ |
| *Serrate* | Sense: 5’-GCTAGGCAGGCGTTTTCC-3’  Antisense: 5’-GCTTAGCGAAGAGAAGAGTTCG-3’ |
| *Notch* | Sense: 5’-TGGATTTTGTGGATCTCCGG-3’  Antisense: 5’-TGAACTTTGTATCGAGGGCG-3’ |
| *Su(H)* | Sense: 5’- CTTGCTGCCGGGTCCTTAC-3’  Antisense: 5’- CTCGCGCATGTACTTCTCCA-3’ |
| *En(spl)* | Sense: 5’-ATGGAATACACCACCAAGACC-3’  Antisense: 5’-GGCGACAAGTGTTTTCAGGTT-3’ |
| *GAPDH* | Sense: 5’- GTCTCCTCTGACTTCAACAGCG -3’  Antisense: 5’- ACCACCCTGTTGCTGTAGCCAA -3’ |
| *NOTCH1* | Sense: 5’- GGTGAACTGCTCTGAGGAGATC-3’  Antisense: 5’- GGATTGCAGTCGTCCACGTTGA -3’ |
| *Jagged1* | Sense: 5’- TGCTACAACCGTGCCAGTGACT -3’  Antisense: 5’- TCAGGTGTGTCGTTGGAAGCCA -3’ |
| *HES1* | Sense: 5’- GGAAATGACAGTGAAGCACCTCC -3’  Antisense: 5’- GAAGCGGGTCACCTCGTTCATG -3’ |

**Table S2. Chromatographic elution conditions**

| **Time (min)** | **Mobile phase A (%)** | **Mobile phase B (%)** |
| --- | --- | --- |
| Initial | 95 | 5 |
| 3 | 75 | 25 |
| 8.5 | 55 | 45 |
| 14 | 5 | 95 |
| 17 | 2 | 98 |
| 17.2 | 95 | 5 |
| 20.0 | 95 | 5 |

**Table S3. Primary growth and invasion location for the *eyeful* tumor assay**

|  | **Primary growth (No Invasion)** | | | | **Invasion** | | | | **Total** | **Invasion Rate** |
| --- | --- | --- | --- | --- | --- | --- | --- | --- | --- | --- |
|  | Ⅰ | Ⅱ | Ⅲ | Total | Head | Thorax | Abdomen | Total |  |  |
| *eyeful* | 45 | 35 | 40 | 120 | 43 | 3 | 3 | 49 | 169 | 28.99% |
| 6.25 Binpu-3LE | 38 | 36 | 47 | 121 | 38 | 6 | 1 | 45 | 166 | 27.11% |
| 12.50 Binpu-3LE | 25 | 21 | 28 | 74 | 21 | 4 | 2 | 27 | 101 | 26.73% |
| 25.00 Binpu-3LE | 49 | 18 | 25 | 92 | 29 | 3 | 0 | 32 | 124 | 25.81% |
| 50.00 Binpu-3LE | 52 | 42 | 56 | 150 | 44 | 8 | 3 | 55 | 205 | 26.83% |
| 6.25 Binpu-3RE | 32 | 18 | 37 | 87 | 18 | 3 | 1 | 22 | 109 | 20.18% |
| 12.50 Binpu-3RE | 30 | 17 | 43 | 90 | 20 | 3 | 3 | 26 | 116 | 22.41% |
| 25.00 Binpu-3RE | 61 | 36 | 40 | 137 | 25 | 3 | 1 | 29 | 166 | 17.47% |
| 50.00 Binpu-3RE | 46 | 28 | 37 | 111 | 31 | 6 | 4 | 41 | 152 | 26.97% |

**Table S4. Identification results of chemical components of base peak chromatograms**

| **Number** | **m/z** | **RT/min** | **ppm** | **Adduct** | **Score** | **Formula** | **Compound Name** | **Content** |
| --- | --- | --- | --- | --- | --- | --- | --- | --- |
| 1 | 166.0864 | 1.99 | 0.2 | [M+H-C11H12N2O2]^+^ | 0.9988 | C_20_H_23_N_3_O_4_ | Gly-Phe-Phe | 0.10% |
| 2 | 220.1184 | 2.18 | 3.2 | [M+H]^+^ | 0.9896 | C_9_H_17_NO_5_ | Pantothenate | 0.08% |
| 3 | 332.1348 | 2.31 | 2.5 | [M+NH4]^+^ | 0.9504 | C_14_H_18_O_8_ | 4-(beta.-D-Glucopyranosyloxy)benzeneacetic acid | 1.39% |
| 4 | 188.0707 | 2.62 | 1.6 | [M+H-H2O]^+^ | 0.972 | C_11_H_11_NO_3_ | 3-Indolyllactic acid | 0.53% |
| 5 | 163.039 | 3.07 | 1.6 | [M+H-C6H6O2]^+^ | 0.9953 | C_15_H_12_O_5_ | Butein | 0.17% |
| 6 | 263.1282 | 3.32 | 1.2 | [M+H]^+^ | 0.9336 | C_15_H_18_O_4_ | 1-Dehydroperuvinine | 0.35% |
| 7 | 229.1227 | 3.42 | 1.6 | [M+H-2H2O]^+^ | 0.8485 | C_15_H_20_O_4_ | Peruvic acid | 0.29% |
| 8 | 425.1812 | 4.31 | 0.8 | [M+H]^+^ | 0.7094 | C_21_H_28_O_9_ | Ncgc00385202-01_c21h28o9_ | 0.78% |
| 9 | 499.1242 | 4.86 | 1.5 | [M+H-H2O]^+^ | 0.9888 | C_25_H_24_O_12_ | 3,5-Dicaffeoylquinic acid | 0.06% |
| 10 | 261.1127 | 5.67 | 2.2 | [M+H-C4H6O3]^+^ | 0.9592 | C_19_H_22_O_7_ | Janerin | 0.08% |
| 11 | 465.1374 | 6.41 | 3.3 | [M+H-H2O]^+^ | 0.9591 | C_24_H_26_O_7_; C_22_H_26_O_12_;  C_28_H_34_O_15_; C_22_H_24_O_11_ | Catalposide | 0.07% |
| 12 | 231.1384 | 6.56 | 1.8 | [M+H-2H2O]^+^ | 0.9274 | C_15_H_22_O_4_ | 3a,8-Dihydroxy-3,5a,9-trimethyl-4,5,6,7,8,9b-hexahydro-3H-naphtho[6,5-d]furan-2-one | 0.34% |
| 13 | 163.0391 | 7.42 | 1 | [M+H-H2O]^+^ | 0.9956 | C_9_H_8_O_4_ | 3-Hydroxy-7-methoxyphthalide | 0.07% |
| 14 | 271.0605 | 8.46 | 1.5 | [M+H]^+^ | 0.9875 | C_15_H_10_O_5_ | Luteolinidin | 0.47% |
| 15 | 343.296 | 11.06 | 1.6 | [M]^+^ | 0.9985 | C_19_H_38_N_2_O_3_; [C_19_H_39_N_2_O_3_]^+^ | Cocamidoprpylbetaine | 0.71% |
| 16 | 518.3247 | 12.75 | 4.3 | [M+H]^+^ | 0.9055 | C_26_H_48_NO_7_P | LPC 18:3 | 2.24% |
| 17 | 520.3404 | 13.36 | 1.3 | [M+H]^+^ | 0.9749 | C_26_H_50_NO_7_P | .beta.-Linoleoyl-.alpha.-glycerophosphorylcholine | 1.65% |
| 18 | 496.3403 | 13.83 | 1 | [M+H]^+^ | 0.9882 | C_24_H_50_NO_7_P | Lyso-PC(16:0) | 0.34% |
| 19 | 522.3563 | 14.05 | 1.7 | [M+H]^+^ | 0.9737 | C_26_H_52_NO_7_P | 18:1 Lyso PC | 0.22% |
| 20 | 398.2332 | 14.45 | 1.7 | [M+H]^+^ | 0.8558 | C_24_H_31_NO_4_ | 2-[4-(Diamylamino)-2-hydroxybenzoyl]benzoic acid | 0.16% |
| 21 | 524.3718 | 14.84 | 2 | [M+H]^+^ | 0.982 | C_26_H_54_NO_7_P | 18:0 Lyso PC | 0.33% |
| 22 | 353.0889 | 2.5 | 2.5 | [M-H]^-^ | 0.953 | C_16_H_18_O_9_ | Neochlorogenic acid | 0.36% |
| 23 | 311.042 | 2.67 | 4.7 | [M-H]^-^ | 0.9804 | C_13_H_12_O_9_ | Caftaric acid | 0.78% |
| 24 | 353.0889 | 3.08 | 3.3 | [M-H]^-^ | 0.9994 | C_16_H_18_O_9_ | Chlorogenate | 1.96% |
| 25 | 473.0742 | 4.41 | 3.3 | [M-H]^-^ | 0.9919 | C_22_H_18_O_12_ | Chicoric acid | 0.16% |
| 26 | 365.089 | 4.96 | 2 | [M-H]^-^ | 0.9643 | C_25_H_24_O_12_ | Rubinaphthin A | 0.14% |
| 27 | 341.0712 | 5.3 | 4.7 | [M-H]^-^ | 0.7121 | C_17_H_18_O_9_ | Benzotript | 0.23% |
| 28 | 187.0981 | 5.83 | 2.7 | [M-H]^-^ | 0.9963 | C_18_H_15_ClN_2_O_3_ | Azelaic acid | 0.17% |
| 29 | 207.067 | 7.32 | 3.5 | [M-H]^-^ | 0.9887 | C_9_H_16_O_4_ | Ethyl trans-caffeate | 0.23% |
| 30 | 327.2189 | 8.8 | 3.4 | [M-H]^-^ | 0.991 | C_11_H_12_O_4_ | (10E,15Z)-9,12,13-Trihydroxyoctadeca-10,15-dienoic acid | 0.82% |
| 31 | 329.2344 | 9.65 | 2.6 | [M-H]^-^ | 0.9932 | C_18_H_32_O_5_ | 9-Octadecenoic acid, 5,8,11-trihydroxy- | 0.13% |
| 32 | 293.1768 | 11.46 | 2.6 | [M-H]^-^ | 0.9504 | C_18_H_34_O_5_ | [6]-Gingerol | 0.51% |
| 33 | 295.2286 | 13.33 | 2.5 | [M-H]^-^ | 0.9952 | C_17_H_26_O_4_ | 12(13)-EpOME | 0.51% |

**Figure S1. HPLC chromatogram of standard controls.**

The standards of four phenolic acids (peaks 1–4 represent caftaric acid, chlorogenic acid, caffeic acid, and cichoric acid) (**a**), three flavonoids (peaks 1–3 represent quercetin, luteolin, and apigenin) (**b**), and two triterpenoids (peaks 1–2 represent taraxerol and taraxasterol) (**c**) were identified using HPLC.

**Figure S2. Binpu-3RE has no effect on food intake.**

Measurement of 20 min food intake by early *w^1118^* third-instar larvae of normal feed or Binpu-3RE medium with different concentrations as calculated using the blue food ingestion method (6–8 larvae per pool, n=3). For food intake statistics, one-way ANOVA with Bonferroni’s multiple comparison test was applied. ns: no significant difference.


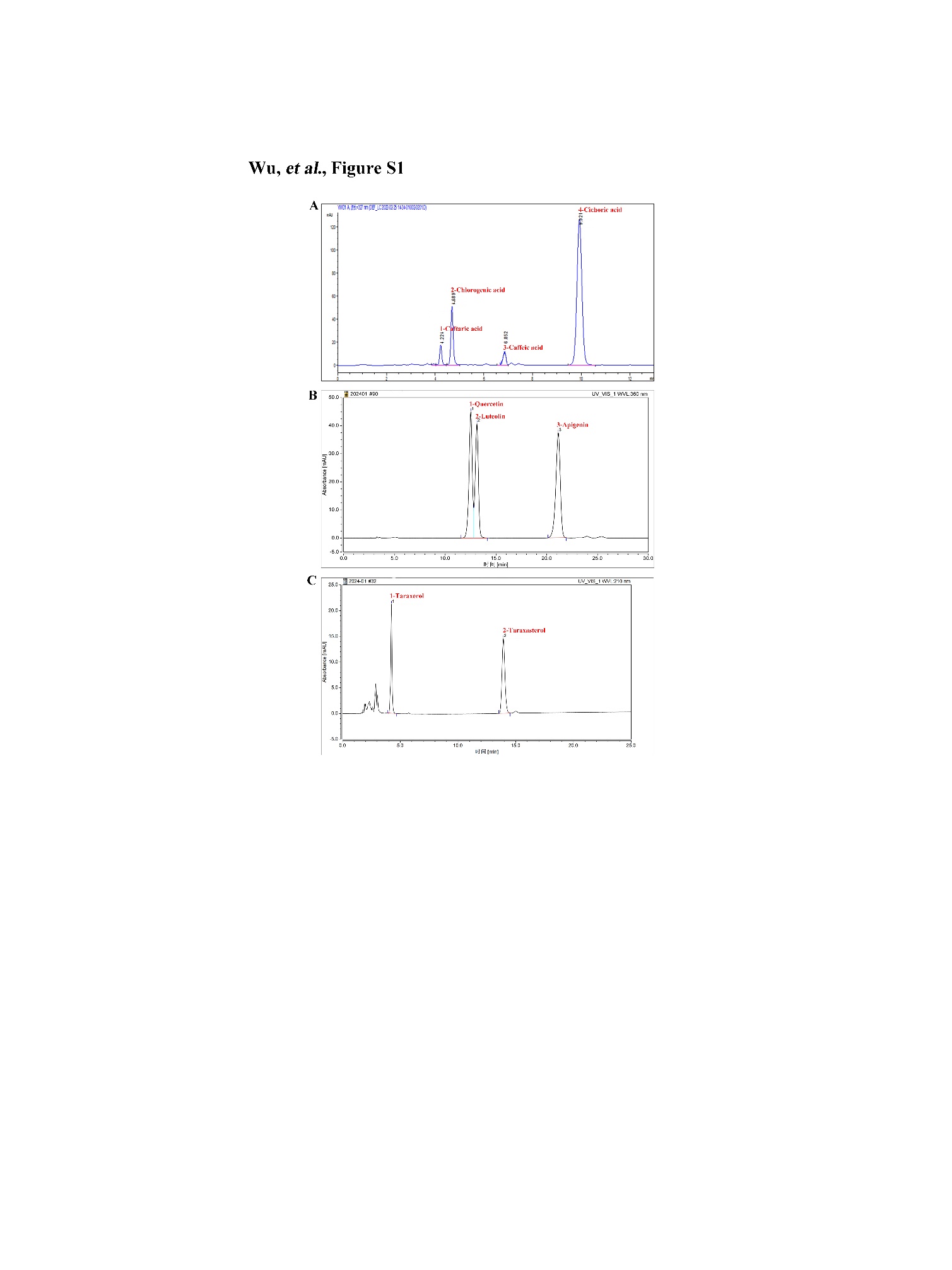

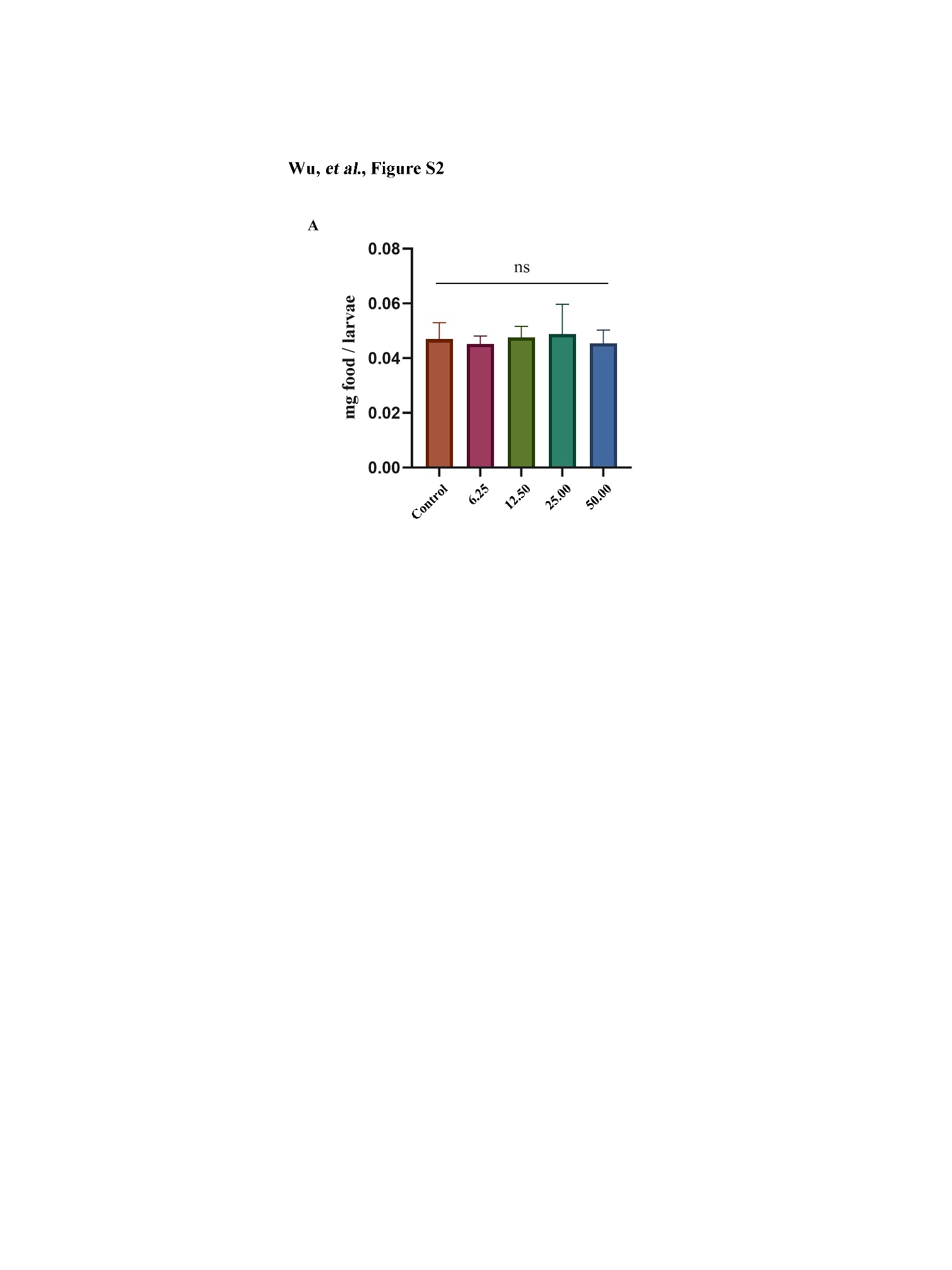

Supplement: Supplementary file 1 [file DataSheet1.docx]
